# Dysregulated fibronectin trafficking by Hsp90 inhibition restricts prostate cancer cell invasion

**DOI:** 10.1038/s41598-018-19871-4

**Published:** 2018-02-01

**Authors:** Heather K. Armstrong, Joanna L. Gillis, Ian R. D. Johnson, Zeyad D. Nassar, Max Moldovan, Claire Levrier, Martin C. Sadowski, Mei Yieng Chin, Emma S. Tomlinson Guns, Gerard Tarulli, David J. Lynn, Douglas A. Brooks, Luke A. Selth, Margaret M. Centenera, Lisa M. Butler

**Affiliations:** 1 Adelaide Medical School and Freemasons Foundation Centre for Men’s Health, University of Adelaide, Adelaide, SA 5000 Australia; 2 South Australian Health and Medical Research Institute, Adelaide, SA 5000 Australia; 3 Mechanisms in Cell Biology and Disease Research Group, School of Pharmacy and Medical Sciences, Sansom Institute for Health Research, University of South Australia, Adelaide, SA 5001 Australia; 4 Australian Prostate Cancer Research Centre - Queensland, Institute of Health and Biomedical Innovation, Queensland University of Technology, Princess Alexandra Hospital, Translational Research Institute, Brisbane, QLD 4102 Australia; 5 The Vancouver Prostate Centre at Vancouver General Hospital, and Department of Urologic Sciences, University of British Columbia, Vancouver, B.C Canada; 6 Dame Roma Mitchell Cancer Research Laboratories, Adelaide Medical School, University of Adelaide, Adelaide, SA 5000 Australia; 7 School of Medicine, Flinders University, Bedford Park, SA 5042 Australia

## Abstract

The molecular chaperone Hsp90 is overexpressed in prostate cancer (PCa) and is responsible for the folding, stabilization and maturation of multiple oncoproteins, which are implicated in PCa progression. Compared to first-in-class Hsp90 inhibitors such as 17-allylamino-demethoxygeldanamycin (17-AAG) that were clinically ineffective, second generation inhibitor AUY922 has greater solubility and efficacy. Here, transcriptomic and proteomic analyses of patient-derived PCa explants identified cytoskeletal organization as highly enriched with AUY922 treatment. Validation in PCa cell lines revealed that AUY922 caused marked alterations to cell morphology, and suppressed cell motility and invasion compared to vehicle or 17-AAG, concomitant with dysregulation of key extracellular matrix proteins such as fibronectin (FN1). Interestingly, while the expression of FN1 was increased by AUY922, FN1 secretion was significantly decreased. This resulted in cytosolic accumulation of FN1 protein within late endosomes, suggesting that AUY922 disrupts vesicular secretory trafficking pathways. Depletion of FN1 by siRNA knockdown markedly reduced the invasive capacity of PCa cells, phenocopying AUY922. These results highlight a novel mechanism of action for AUY922 beyond its established effects on cellular mitosis and survival and, furthermore, identifies extracellular matrix cargo delivery as a potential therapeutic target for the treatment of aggressive PCa.

## Introduction

Prostate cancer (PCa) is the second leading cause of cancer-related deaths, and the most commonly diagnosed malignancy in Western men^1,2^. Early diagnosis of localized disease facilitates effective treatment using radiation or surgery, but for 20–30% of men these therapies are not curative^3^. A hallmark of PCa cells is their critical dependence on androgen signaling, and the androgen receptor (AR) is the primary therapeutic target for relapsed or advanced disease^4,5^. However, therapy resistance is inevitable, and more recent treatment options such as the AR antagonist enzalutamide^6^ and the CYP17 inhibitor abiraterone acetate^7^ achieve only limited survival benefits. Consequently, there is an urgent need for new therapeutic options to significantly improve survival outcomes.

The molecular chaperone Hsp90 regulates the stabilization, maturation and activation of over 200 client proteins, including the AR^8,9^. As many Hsp90 clients are known oncoproteins, cancer cells have a greater dependence on Hsp90 for growth and survival compared to non-malignant cells^10–12^. This dependence is further exacerbated by the increased number of mutated or misfolded proteins known to accumulate within cancer cells, as these are reliant on Hsp90 to prevent their degradation^8,13^. Moreover, upregulation of Hsp90 is a common feature of many tumor cell types including PCa, making it a potentially selective target for cancer therapy^8,13^.

Despite promising preclinical efficacy, first-in-class Hsp90 inhibitors such as the geldanamycin derivative 17-allylamino-demethoxygeldanamycin (17-AAG) have proven to be largely disappointing in clinical trials, reviewed in^14^. Next generation inhibitors, including synthetic small molecules such as AUY922, possess increased potency and more favorable pharmacological properties^15^, suggesting that they may be clinically more efficacious. Using patient-derived prostate tumor tissues, cultured as explants, we previously demonstrated that AUY922 has greater biological activity than 17-AAG in terms of reducing tumor cell proliferation and inducing apoptosis^16^. An important observation from that study was that both 17-AAG and AUY922 significantly induced the expression of Hsp70, a clinically-used marker of Hsp90 inhibition, whereas only AUY922 was capable of significantly reducing proliferation and inducing apoptosis^16,17^. The downstream mechanisms that differentiate the relative efficacies of next generation versus first-in-class HSP90 inhibitors remain unclear. This study identified pathways selectively altered by AUY922, and not 17-AAG, in patient-derived PCa explants and further interrogated the influence of those pathways on the anti-tumor activity of AUY922.

## Results

### Cytoskeletal organization pathways are selectively altered by AUY922 in patient-derived prostate explants

**Figure 1:**
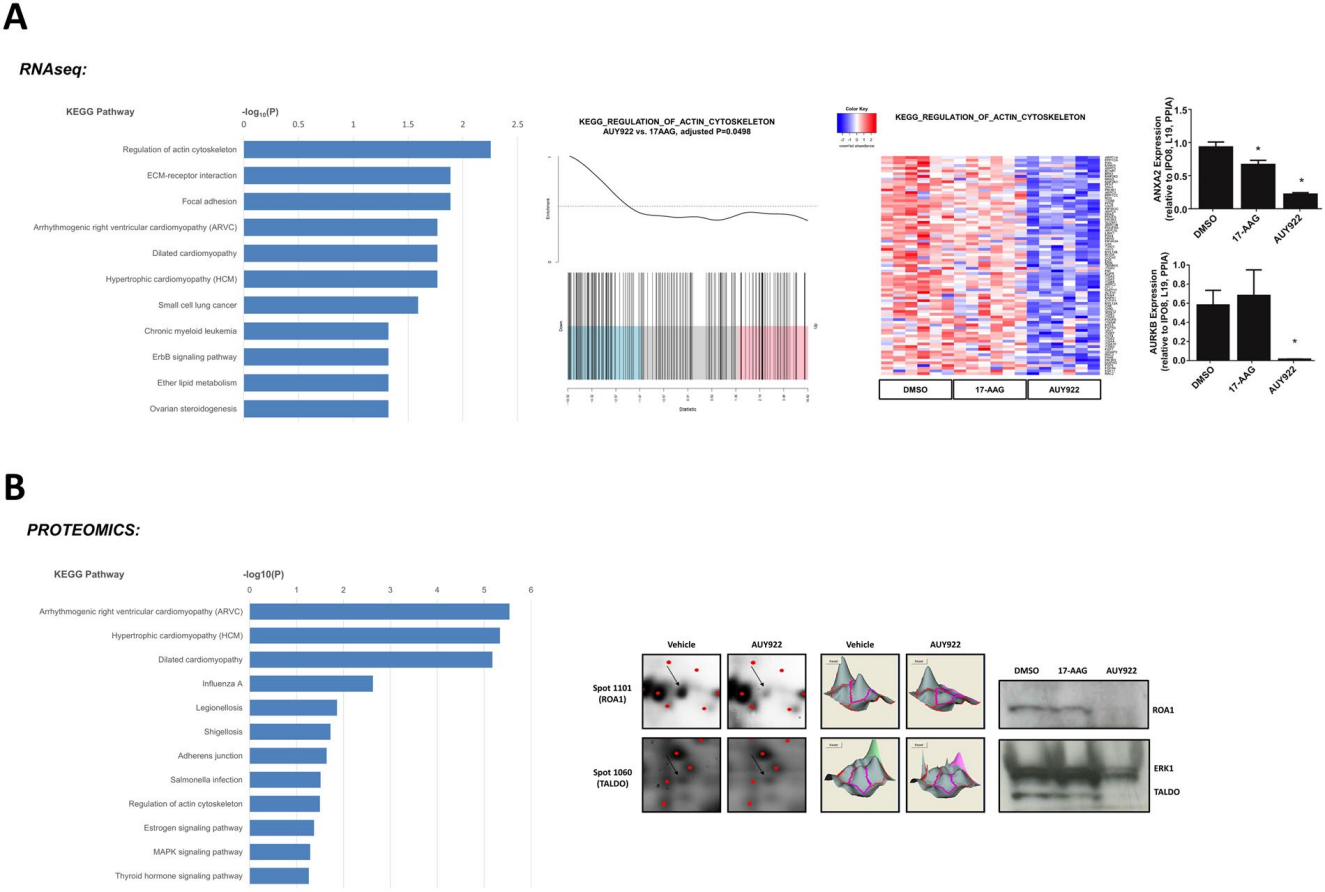
The Hsp90 inhibitor, AUY922, selectively alters cellular pathways involved in the cytoskeleton and ECM. (**A**) KEGG pathways determined by over-representation analysis of differentially expressed genes identified through RNA-seq analysis of AUY922 treated PDEs (48 h: 500 nM) using the InnateDB database. A barcode plot and heatmap of RNA-seq gene expression data from regulation of actin cytoskeleton pathway for individual explants is shown, and significant changes in gene expression were validated in independent explants by qRT-PCR (ANXA2 & AURKB shown). (**B**) KEGG pathways determined by the Database for Annotation, Visualization and Integrated Discovery (DAVID) using differentially expressed proteins identified through proteomic analysis of AUY922 treated PDEs 48 h: 500 nM, n = 12). Proteins identified by 2D-DIGE proteomics analysis were validated in independent PDE samples by Western blot. ERK1 was used as loading control. Full-length blots are presented in Supplementary Figure 5.

We have previously demonstrated superior efficacy of a second generation (AUY922) versus a first generation (17-AAG) Hsp90 inhibitor in PCa cell lines and patient-derived prostate tumor explants, despite similar induction of the clinically used biomarker Hsp70. To identify novel gene and protein pathways that may underpin this differential anti-proliferative response, patient-derived PCa explants (PDEs) cultured with each agent or vehicle alone were analyzed by transcriptomic (RNA-seq, n = 6 patients) and proteomic analyses (n = 12 patients). As previously reported^16^, we observed enhanced anti-proliferative effects of AUY922 in both prostate cancer PDE cohorts (Supplementary Figure 1). RNA-seq analysis identified 1698 differentially expressed genes (DEGs; p < 0.05) in AUY922 treated PDEs compared with vehicle treatment and 715 DEGs (p < 0.05) compared to 17-AAG treated PDEs, see Supplementary Dataset for DE analysis outcomes. At a pathway level, the KEGG pathways enriched by AUY922 in the RNA-seq dataset revealed Regulation of Actin Cytoskeleton and Extracellular Matrix (ECM) interactions to be the most robustly altered pathways (Fig. 1A). Gene set enrichment analysis demonstrates a significant negative association between AUY922 treatment and Regulation of Actin Cytoskeleton (Fig. 1A) and ECM interactions (Supplementary Figure 2) pathways and heat maps depict genes from both pathways that are exclusively and significantly inhibited by AUY922 (Fig. 1A, Supplementary Figure 2). We validated differential regulation of two genes, *ANXA2* and *AURKB*, by qPCR in independent explants (Fig. 1A). 2D-DIGE proteomic analysis identified 137 protein spots as differentially expressed in AUY922 treated explants compared with vehicle treatment (p < 0.05) and 98 differentially expressed protein spots compared to 17-AAG (p < 0.05). Proteins of interest were those that were differentially expressed in AUY922 treated PDEs compared with both vehicle and 17-AAG, resulting in 26 proteins spots being subjected to identification by mass spectrometry. Pathway analysis using the Database for Annotation, Visualization and Integrated Discovery (DAVID)^18,19^ again revealed Regulation of Actin Cytoskeleton as a highly enriched KEGG pathway in AUY922 treated tumors (Fig. 1B), validating our RNAseq pathway analysis. We validated two proteins, TALDO and ROA1, as being selectively regulated by AUY922 in independent explant tissues (Fig. 1B).

**Figure 2:**
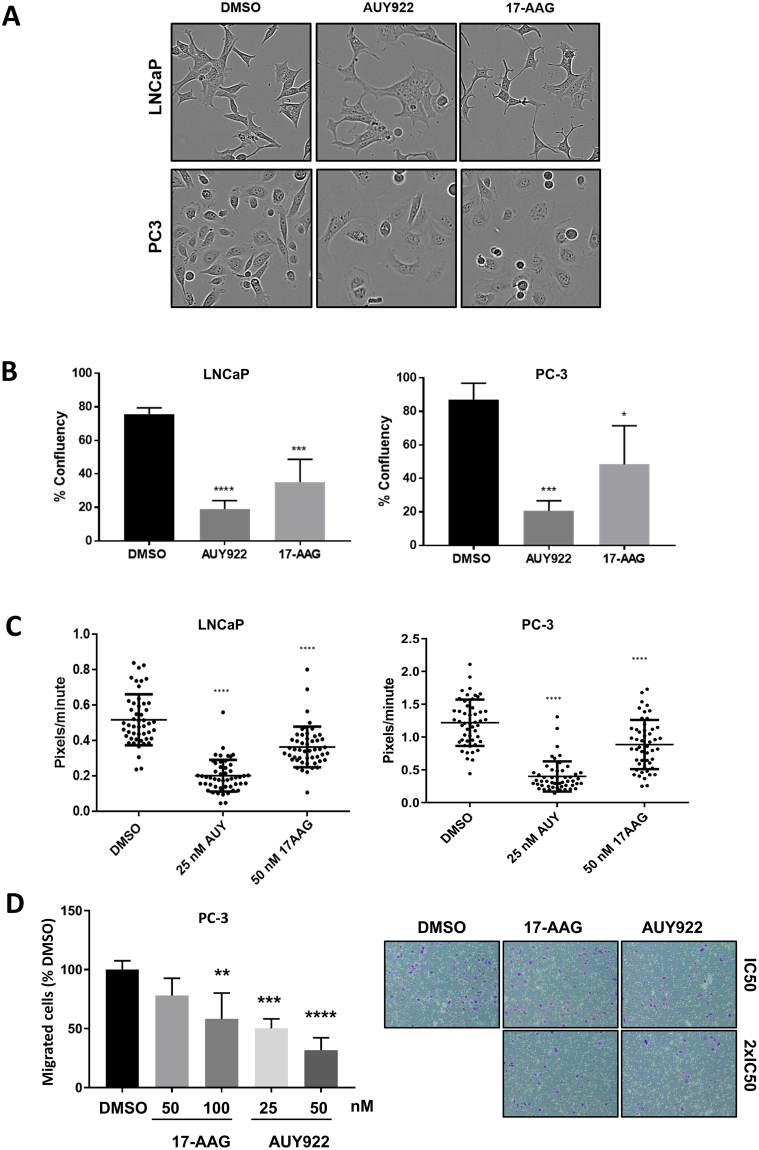
Hsp90 inhibition markedly influences cell morphology, growth, motility and invasive capacity. (**A**) Morphology of LNCaP and PC-3 cells cultured +/− AUY922 (25 nM) or 17-AAG (50 nM) for 48 h in the Incucyte live cell analysis system. (**B**) Cell confluency was measured in the same experiment. (**C**) Motility of LNCaP and PC-3 cells cultured +/− AUY922 (25 nM) or 17-AAG (50 nM) over a 48 h incubation in an Incuyte live cell analysis system, with 50 cells tracked per treatment. (**D**) PC-3 cells were cultured with either IC50 or 2x IC50; AUY922 (25 and 50 nM) or 17-AAG (50 and 100 nM) or vehicle (DMSO) for 48 h before harvesting for a 3D invasion assay using 24-well Falcon BioCoat Matrigel invasion chambers. Migrated cells remaining on the bottom surface were fixed in formalin and stained with crystal violet. Five random fields for each membrane were photographed using the Axio Scope A1 Fluorescent Microscope (Zeiss), and the number of migrated cells was counted manually and presented as percent of control cells ± SEM.

The cytoskeleton makes up the structural framework of the cell, thereby governing cell shape and cytoplasmic organization while regulating cellular functions such as division and motility. The effect of AUY922 on cytoskeletal structure was further reflected in the dramatic morphological changes, including markedly increased cell body size (Fig. 2A) that accompanied an overall decrease in cell number, as measured by confluency (Fig. 2B). Given the pathways differentially altered by AUY922 compared to 17-AAG in the proteomic and transcriptomic analyses, the effects of both drugs on cell motility and invasion through Matrigel were compared in PCa cell lines, using equivalent effective doses of each compound with respect to cell viability (IC50). By tracking cell movement over a 48 hour period post-treatment, a significant and rapid decrease in motility of PC-3 and LNCaP cells (Fig. 2C) and invasion of PC-3 cells (dose-dependent; Fig. 2D) was observed in the presence of AUY922, but to a lesser extent 17-AAG.

### Fibronectin is induced in response to AUY922 and accumulates within cytosolic compartments

**Figure 3:**
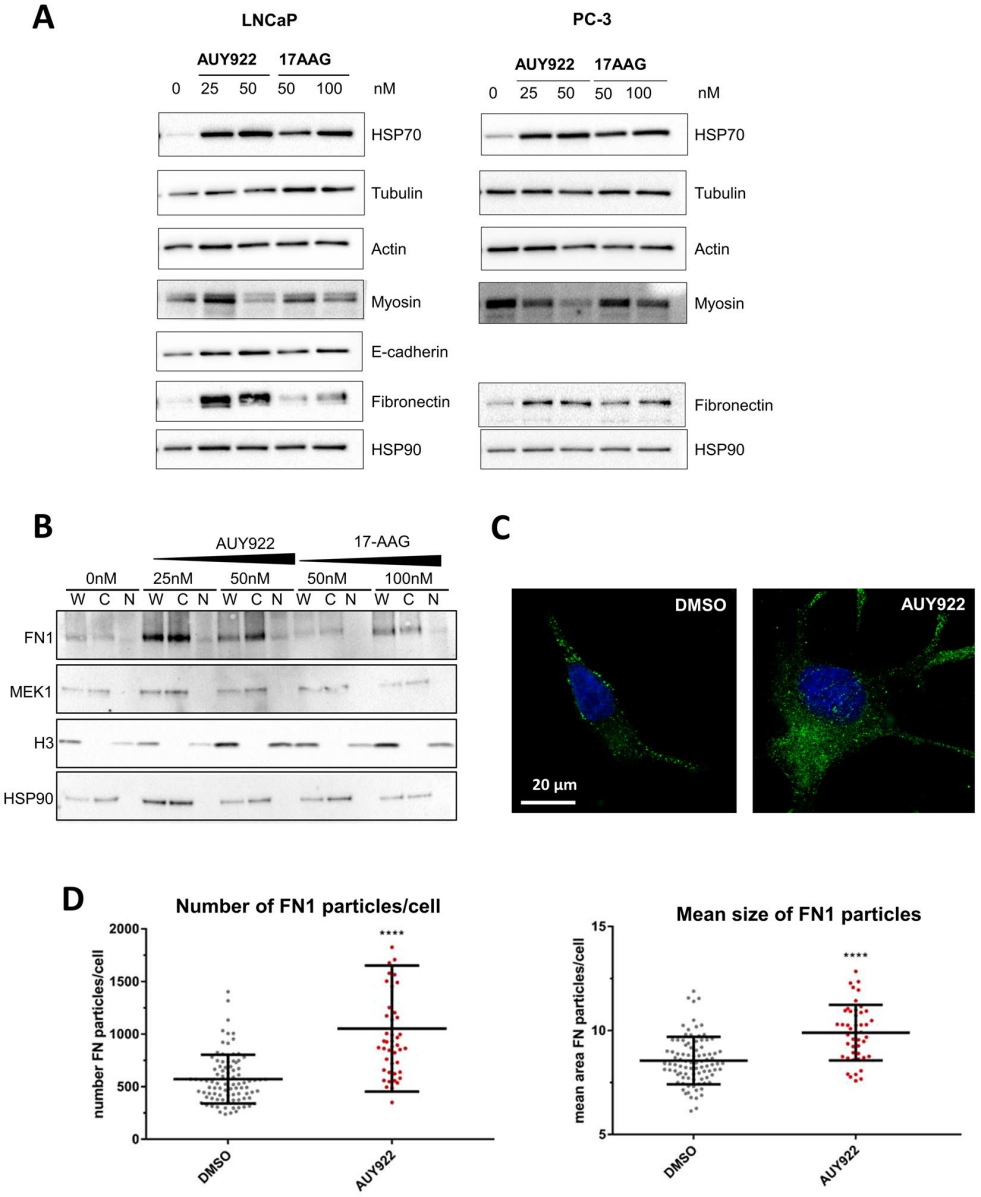
AUY922 induces expression of fibronectin, resulting in its cytosolic accumulation in punctate compartments. (**A**) Western blot analysis of the effect of 17-AAG or AUY922 on Hsp70, fibronectin (FN1) and cytoskeletal proteins, in LNCaP and PC-3 cells. Cells were treated with DMSO control or the indicated doses of AUY922, or 17-AAG and collected at 48 h. Full-length blots are presented in Supplementary Figure 6. To examine intracellular localization of FN1, we used **(B**) cellular fractionation of LNCaP cells treated as indicated for 48 h (W = whole cell lysate, C = cytoplasmic fraction, N = nuclear fraction; full length blots are presented in Supplementary Figure 6), and **(C**) fluorescence microscopy of LNCaP cells treated for with AUY922 (25 nM) for 48 h and probed for FN1 (antibody obtained from Professor Jane Sottile, Rochester University) followed by secondary ALEXA 488 with images taken using Leica SP8 confocal microscope at 40x magnification. (**D**) The number and distribution of FN1 particles in cultured LNCaP cells with AUY922 (25 nM) for 48 h was quantified by automated image analysis (CellProfiler) of fluorescence microscopy images captured with an INCell 2200 automated imaging system (GE Healthcare) at 20x magnification.

Based on its pronounced effects on cytoskeletal pathways, prostate cell motility and morphology, a panel of cytoskeletal and ECM-related proteins was assessed in LNCaP and PC-3 cells cultured for 48 h with IC50 and 2x IC50 concentrations of AUY922 (25 nM, 50 nM) or 17-AAG (50 nM, 100 nM) (Fig. 3A). Hsp90 inhibition was confirmed by expression of the clinical biomarker Hsp70, which was induced in response to both AUY922 and 17-AAG in LNCaP and PC-3 cells (Fig. 3A). Also in both cell lines, AUY922 did not change expression of tubulin or actin relative to vehicle treatment, but decreased the amount of myosin motor protein in a dose dependent manner (Fig. 3A). Both agents enhanced the expression of E-cadherin in LNCaP cells but AUY922 did so to a greater extent; E-Cadherin was not expressed in PC-3 cells. The most notable effect of AUY922 was a marked induction of fibronectin (FN1) protein compared to 17-AAG treatment in both cell lines (Fig. 3A). The AUY922-selective induction of FN1 observed in PCa cell lines, along with its presence in the ECM and cytoskeletal KEGG pathways that were enriched in out transcriptomic data from the PDE tissues (Fig. 1A, Supplementary Figure 2), led us to focus on this protein.

Whole cell, cytosolic and nuclear fractions from LNCaP cells indicated that FN1 accumulated within the cytosol following 48 h treatment with AUY922 and, to a lesser extent, with 17-AAG (Fig. 3B). Immunofluorescent staining showed that although FN1 remained localized to the same cellular compartments in AUY922 treated cells compared to vehicle (Fig. 3C), quantitative analysis of the FN1 staining in multiple cells revealed significant AUY922-dependent increases in both the number and size of FN1-containing particles (Fig. 3D).

### AUY922 inhibits fibronectin secretion

**Figure 4:**
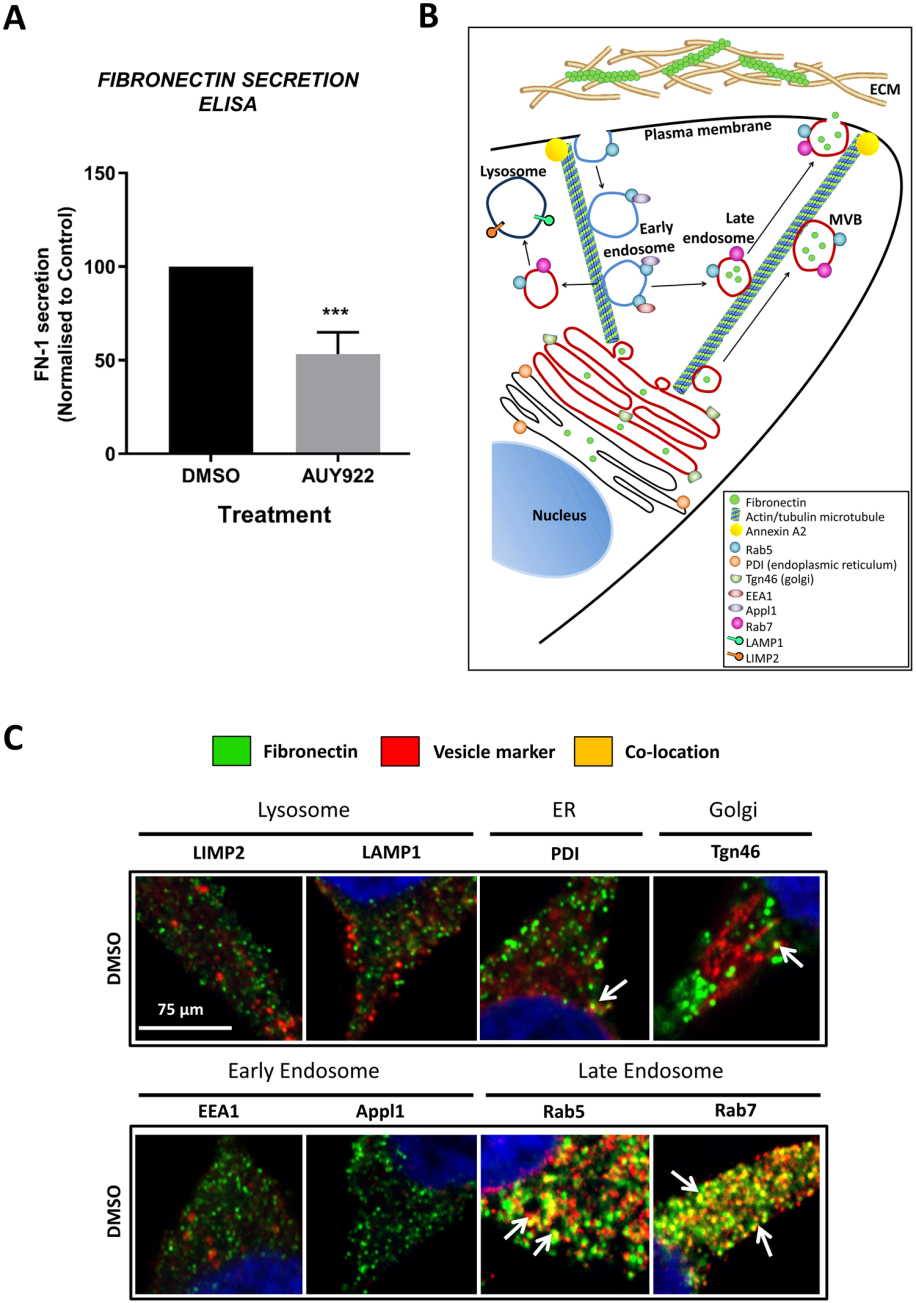
Fibronectin is localized to late endosome compartments which require intact tubulin microtubules to traffic to the plasma membrane. (**A**) To examine inhibitor effects on secretion of FN1, an ELISA assay was performed on tissue culture media (HPC-1) collected from LNCaP cells treated with AUY922 (25 nM) for 48 h and controlled for cell number. Date represents mean ± SEM of  4 independent experiments. (**B**) Model illustrating cytosolic vesicle and compartment trafficking to the plasma membrane along microtubule networks. (**C**) Co-location of FN1 with vesicle markers including endosomes (EEA1, Appl1, Rab5, Rab7), lysosome (LIMP2, LAMP1), endoplasmic reticulum (PDI) and Golgi (Tgn46) was examined by fluorescence microscopy in LNCaP cells treated with DMSO for 48 h and probed for vesicle markers (red) and FN-1 (green) and DAPI mount media (blue). Images were taken using Leica SP8 confocal microscope at 63x magnification.

The function of FN1 is largely considered to rely upon its secretion from the cell and subsequent incorporation into the ECM^20,21^. However, despite the marked induction of FN1 expression in AUY922 treated PCa cells, ELISA assays performed to detect FN1 in the culture medium demonstrated that AUY922 (25 nM) caused a consistent and significant reduction in FN1 secretion by LNCaP cells compared to vehicle treated cells (Fig. 4A; n = 4 independent experiments).

The cellular secretion of proteins is reliant on endosome traffic along microtubules for delivery to the plasma membrane, where the release of ECM proteins including FN1 occurs, allowing for their polymerization and deposition in the extracellular space (Fig. 4B). To determine which cytosolic compartments contained FN1, co-staining of FN1 was performed in LNCaP cells with a panel of endoplasmic reticulum, Golgi, endosome and lysosome vesicle markers including PDI, Tgn46, Appl1, EEA1, Rab5, Rab7, LIMP2 and LAMP1 (Fig. 4C). FN1 co-located with PDI (endoplasmic reticulum), Tgn46 (Golgi) and, most robustly, vesicles containing the Rab5 (early endosomal) and Rab7 (late endosomal) markers (Fig. 4C). Visual inspection suggests that colocation of FN1 with these markers was unaltered by AUY922 (Supplementary Figure 3).

### Suppression of fibronectin secretion is not due to disrupted microtubules or exosome release

**Figure 5:**
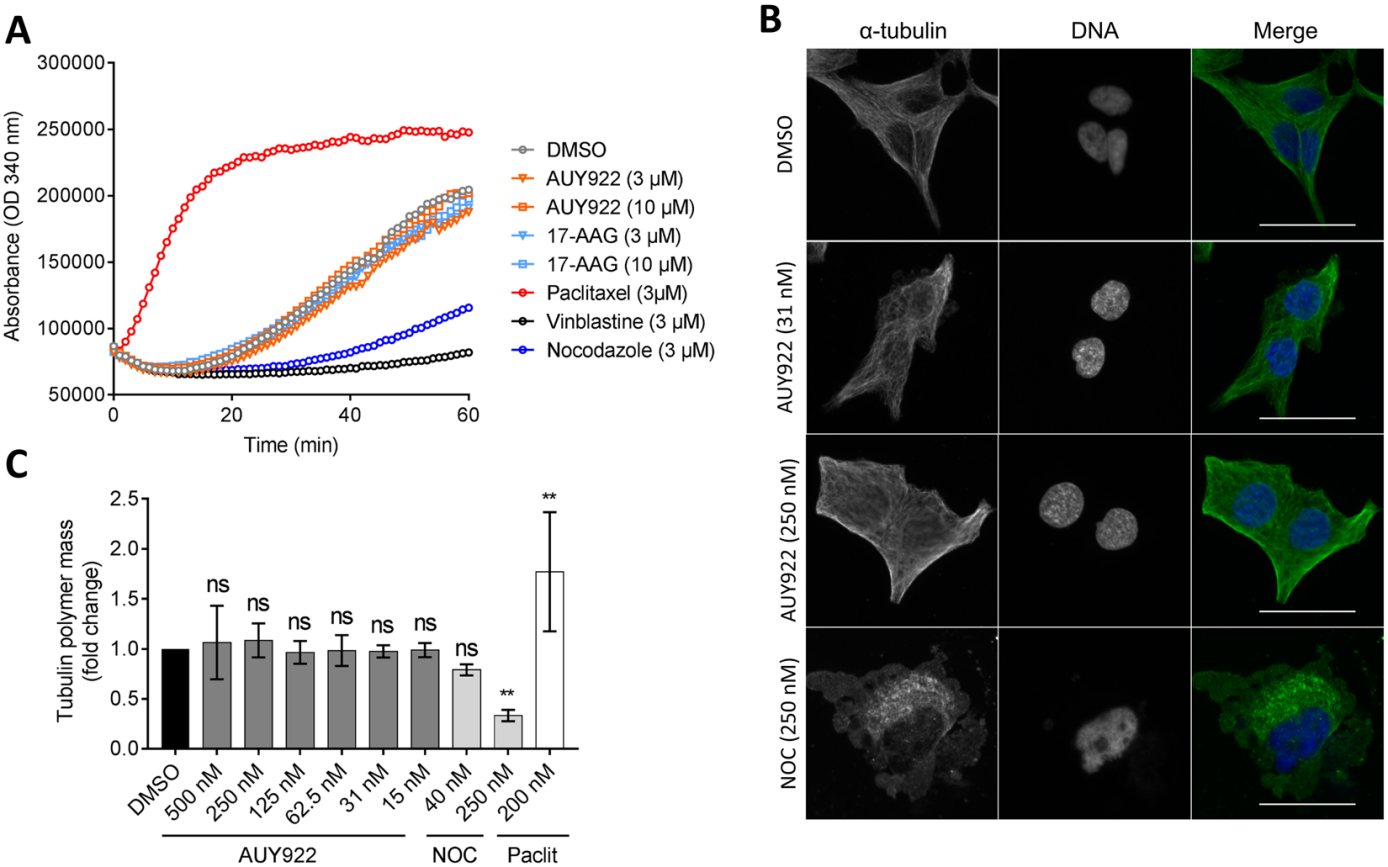
AUY922 does not affect microtubule polymerization in prostate cancer cells. (**A**) AUY922, 17-AAG and control compounds at the indicated doses were incubated with purified tubulin protein at 37 °C, and polymerization was monitored using a FLUOstar Omega plate reader at 340 nm every 1 min for 60 min at 37 °C. (**B**) Representative images of LNCaP cells treated with AUY922 (31 or 250 nM), nocodazole (NOC) (250 nM) or vehicle control (DMSO) for 48 h and subjected to fluorescence microscopy of α-tubulin (green) and DNA (blue) using an INCell 2200 automated imaging system at 40x magnification. Cells treated with DMSO or AUY922 showed intact microtubule network, while cells treated with the microtubule destabilizing drug NOC displayed depolymerized microtubules. Scale bar = 5 µm. (**C**) AUY922 (15–500 nM) did not affect α-tubulin polymer mass, while NOC (250 nM) and paclitaxel (200 nM) reduced and increased, respectively, α-tubulin polymer mass, as shown by quantitative immunofluorescence microscopy of the cellular α-tubulin mean intensity as depicted in (**B**) (expressed as fold-change relative to DMSO control, n = 3, mean ± SD).

To further investigate the mechanism of impaired FN1 secretion, the effect of AUY922 on microtubule function was evaluated using a tubulin polymerization assay with purified tubulin (Fig. 5A). As expected, the microtubule-destabilizing drugs vinblastine and nocodazole inhibited microtubule polymerization, whereas the microtubule-sensitizing drug paclitaxel increased microtubule polymerization (Fig. 5A). No direct effect of the Hsp90 inhibitors on tubulin polymerization was detected (Fig. 5A). Moreover, no disruption of the microtubule network was evident in LNCaP cells treated with AUY922 (Fig. 5B), nor any changes to the cellular α-tubulin polymer mass (Fig. 5C).

An alternative hypothesis was that AUY922 may affect FN1 secretion via impaired exosome release, as FN1 is a reported exosomal cargo protein in PCa cell lines and patient serum^22,23^. However, there was no consistent effect of AUY922 on exosome release in 3 independent cell lines (LNCaP, PC-3 and 22Rv1) (Supplementary Figure 4), and the FN1 content of those exosomes was only modestly reduced by AUY922 (Supplementary Figure 5). 

### Depletion of cellular fibronectin suppresses PCa cell invasion to a similar extent as Hsp90 inhibition

**Figure 6:**
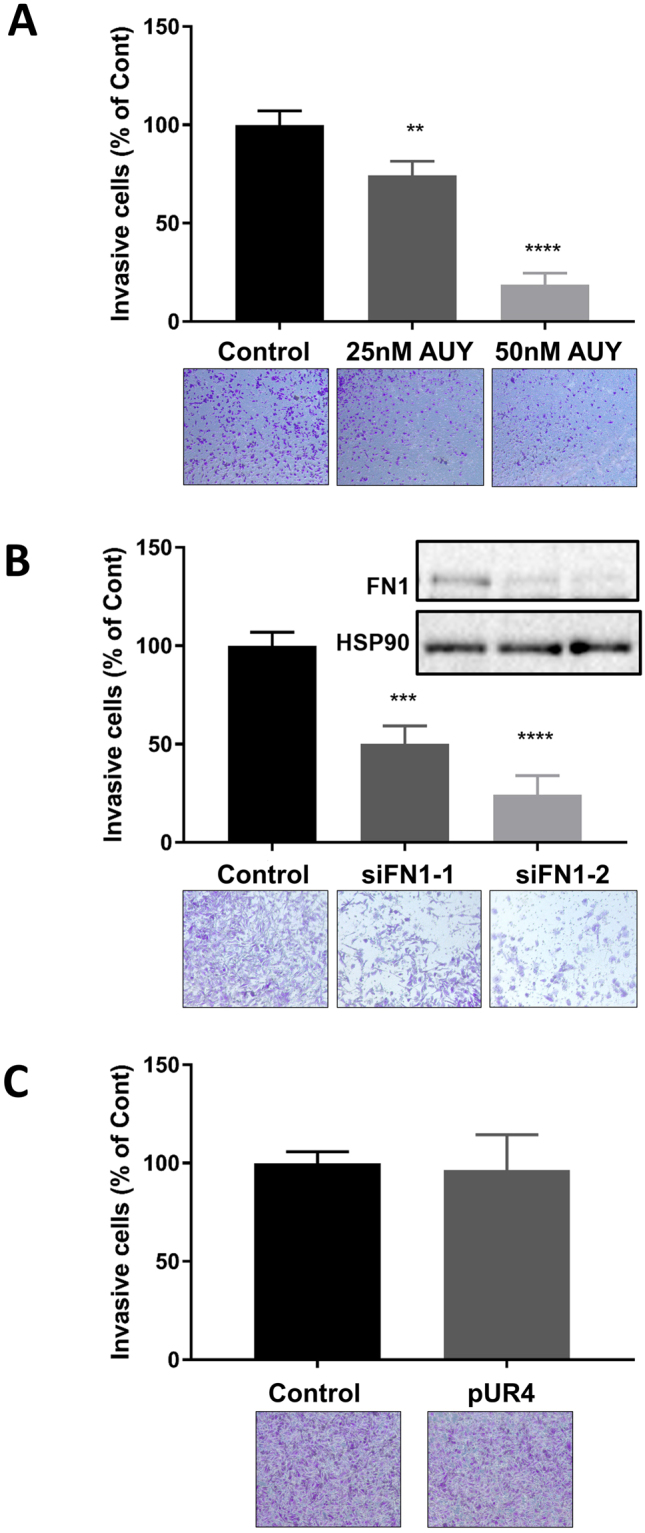
AUY922 and specific knockdown of FN1 both suppress prostate cancer cell invasion. (**A**) Cells were cultured with either AUY922 (25, 50 nM), or vehicle (DMSO) for 48 h before harvesting for the invasion assay. (**B**) PC-3 cells were transfected with 2 independent siRNAs against FN1 (Si-1, Si-2), or non-targeting siRNA as a negative control for 48 h before harvesting for the invasion assay. (**C**) PC-3 cells were treated with pUR4 (500 nM) or the control peptide III-11C (500 nM) for 48 h before harvesting for the invasion assay. 3D invasion assays were conducted in PC-3 cells using 24-well Falcon BioCoat Matrigel invasion chambers. Treated or siRNA-transfected cells were seeded into the upper chamber of the Transwell at a density of 1x 10^5^ cells/well in serum-free medium. The bottom chamber was filled with RPMI-1640 medium containing 5% FBS and inserts incubated at 37 °C for 48 h. Migrated cells remaining on the bottom surface were fixed in formalin and stained with crystal violet. Five random fields for each membrane were photographed using the Axio Scope A1 Fluorescent Microscope (Zeiss), and the number of migrated cells was counted manually and presented as percent of control cells ± SEM.

We next investigated whether cellular accumulation or secretion of FN1 was biologically meaningful in the efficacy of AUY922 to suppress invasion and migration, or merely a marker of general cytoskeletal and/or secretory processes being altered by Hsp90 inhibition. As secretion of FN1 has been implicated as a key mediator of tumor cell invasion and migration, and we found Hsp90 inhibition to suppress FN1 secretion and tumor cell invasion (Fig. 2D), a Matrigel invasion assay was employed in PC-3 cells to compare the effects of AUY922 to (a) siRNA knockdown of FN1 causing depletion of cellular and secreted FN1, or (b) inhibition of FN1 polymerization and ECM fibril deposition using the inhibitory peptide pUR4^24^. Significant, dose-dependent, inhibition of PC-3 cell invasion was observed using AUY922 (Fig. 6A). Knockdown of FN1 expression by approximately 80% was achieved using 2 independent siRNAs, and both caused a significant inhibition of PC-3 cell invasion, to a similar degree as AUY922 (Fig. 6B). In contrast, there was no significant effect of the pUR4 inhibitory peptide on cell invasion (Fig. 6C), suggesting that fibril deposition of FN1 is not required for invasiveness.

## Discussion

The Hsp90 inhibitor AUY922 exhibits potent anti-tumor activity in a variety of cancers, including PCa, which is the second leading cause of cancer related deaths for men worldwide^25–29^. This study aimed to identify the cellular and molecular mechanisms that contribute to the enhanced efficacy of this agent compared to the first-in-class inhibitor 17-AAG, an outcome that could facilitate development of new and improved therapeutics and/or identify more appropriate biomarkers of the Hsp90 inhibitor response. Using a transcriptomic approach coupled with proteomic analysis of a unique patient-derived explant model, we provide evidence for a novel effect of AUY922-mediated inhibition of Hsp90 function leading to altered cytoskeletal organization that is associated with changes in fibronectin expression, intracellular delivery and secretion, and is consistent with an impact on PCa cell migration and metastatic potential.

Cytoskeletal organization and microtubule dynamics have been associated with tumor cell dissemination and metastasis in multiple studies and model systems^30–33^. Changes in cytoskeletal organization, often driven via epithelial to mesenchymal transition (EMT), can mediate increased cancer cell motility, invasion and metastasis^30,31,34^. Fibronectin is a core glycoprotein component of the extracellular matrix that plays an important role in the maintenance and regulation of cell structure, cell adhesion, physiological remodeling, proliferation, differentiation and survival^35^. Most often in the study of cancer, fibronectin is examined as a marker of the mesenchymal phenotype associated with EMT for the purpose of metastasis^36^, or is considered primarily in the context of fibroblastic stroma in the tumor microenvironment. Much less is known about the potential roles that fibronectin may play in normal and malignant epithelium. However, loss of fibronectin expression can contribute to reduced adhesion and other morphological changes in tumor cells^37^. In this study, we discovered that fibronectin secretion is markedly reduced in cells treated with AUY922, despite increased expression and accumulation in late endosome compartments within the cytosol. Our functional studies demonstrated that siRNA depletion of fibronectin expression recapitulated the effects of AUY922 on PCa cell invasion. This led us to speculate that reduced fibronectin secretion, rather than the aberrant intracellular accumulation of fibronectin, underlies the anti-metastatic effects of AUY922 and is an important area for future investigation.

Although little is currently understood about the mechanistic basis of fibronectin secretion, one previous study has suggested that trafficking of fibronectin-containing vesicles occurs via endosome/lysosome pathways and that branched microtubule assembly is required for such trafficking to occur^38^. Our demonstration that fibronectin has a punctate pattern of expression within PCa cells, and co-localizes with PDI, Tgn46, Rab5 and Rab7, suggests that fibronectin is localized within vesicles in the ER, Golgi, early endosomal and late endosomal compartments. The increase in the number and size of fibronectin-containing particles, toether with the enhanced cytoplasmic content of fibronectin, in response to AUY922 treatment likely reflects the accumulation of fibronectin-containing vesicles within the cellular cytoplasm. The GTPase Rab7 is localized to the late endosome and is involved in directing proteins from the early endosome either via multi-vesicular bodies (MVB) to lysosome compartments for degradation or to the cell surface for extracellular release^39–41^. Co-location of fibronectin to these Rab7 endosomes and not the lysosome (LAMP1) suggests that the mechanism of fibronectin trafficking and secretion occurs via Rab7 late endosome and MVB exocytosis pathways. Most attention in the literature has focused on the integrin-dependent secretion of fibronectin into the extracellular space prior to its polymerization and deposition into a fibrillary matrix. More recently, fibronectin has been detected in association with extracellular vesicles released from PCa cell lines and in plasma and urine from clinical patients^22,23^, suggesting an alternative mechanism of cellular release of fibronectin and other matrix-related factors. Importantly, not only can extracellular vesicles promote motility and migration of tumor cells^42^, their cargo of extracellular matrix components, and specifically fibronectin located on the outer surface of these vesicles, have been implicated as a key mediator of directional invasive capacity, potentially due to stabilization of tumor cell protrusions into the stroma and tumor microenvironment^43^, and/or inter-cellular cross-talk^44^. While attractive as a mechanism for the suppressed fibronectin secretion in AUY922-treated cells, we detected no consistent change in extracellular vesicle release from multiple PCa cell lines cultured with AUY922, indicating that the defect likely lies either in the integrin-mediated release of MVBs at the plasma membrane or with intracellular trafficking along the microtubule network.

Vesicle trafficking to the plasma membrane is dependent on polymerized microtubule structures and microtubule-associated proteins^45,46^, and several cytoskeletal proteins including actin and tubulin are known client proteins of Hsp90^47–51^. Inhibition of either fibronectin secretion or tubulin microtubule assembly has been linked to suppression of adhesion^52^, apoptosis^53–55^, metastases^56,57^ and proliferation^54^ of tumor cells, and we therefore hypothesized that Hsp90 maintains the homeostasis of the microtubule network that provide the framework for trafficking of secretory vesicles. Our detailed analysis of both direct and indirect effects of AUY922 on microtubule polymerization and structure revealed no significant effects. We did however observe a marked reduction in myosin motor protein expression in response to AUY922, which could indicate that the process of vesicular traffic is altered and that this impacts on the delivery of endocytic cargo, thereby reducing secretion of fibronectin and promoting its observed accumulation in late endosomes within the enlarged cytosol. Following AUY922 treatment, the majority of fibronectin was detected in Rab5 and Rab7 endosomes and the size and number of fibronectin particles increased in PCa cells, consistent with the intracellular accumulation of fibronectin aggregates in endosomes.

In summary, this study has defined the extracellular matrix component fibronectin as a new cellular target for the Hsp90 inhibitor, AUY922, in PCa cells. Importantly, we provide the first evidence that selective suppression of fibronectin secretion significantly inhibits the invasive capacity of prostate cancer cells, and AUY922 could therefore have potent anti-metastatic action through this pathway. Moreover, as a secreted protein, detection of fibronectin in patient serum may be a useful marker of AUY922 activity *in vivo*. More broadly, we propose that defining the cellular responses to cancer therapeutics using a clinically-relevant tumor explant model is a powerful new approach to not only glean new insights into drug action, but also identify clinically-relevant biomarkers that may indicate patient responsiveness and potential new mechanisms of resistance. Here we show that molecular chaperone cargo is a new therapeutic target in PCa, where the inhibition of cargo delivery can limit the invasive, metastatic potential of aggressive cancer cells.

## Materials and Methods

### Cell culture and reagents

The human prostate carcinoma cell lines 22Rv1, LNCaP, and PC-3 were obtained from the American Type Culture Collection (ATCC). Cell Bank Australia performed verification of all cell lines in 2016 via short-tandem repeat profiling. The 22Rv1 and LNCaP cell lines were maintained in RPMI-1640 medium containing 10% fetal bovine serum (FBS; Sigma Aldrich). The PC-3 cell line was maintained in RPMI-1640 medium containing 5% FBS. The Hsp90 inhibitors 17-N-allylamino-17-demethoxygeldanamycin (17-AAG; National Cancer Institute) and AUY922 (Novartis, now Vernalis) were dissolved in dimethyl sulfoxide (DMSO; Sigma Aldrich). The tubulin modifying drugs paclitaxel and nocodazole (Sigma Aldrich) were dissolved in DMSO. The sources and experimental conditions for primary antibodies used in this study are detailed in Supplementary Table 1.

### *Ex Vivo* Culture of Human Prostate Tumors

Ethical approval for the use of human prostate tumors was obtained from the Ethics Committees of the University of Adelaide (Adelaide, Australia), and St Andrew’s Hospital (Adelaide, Australia). All experiments were performed in accordance with the guidelines of the National Health and Medical Research Council (Australia). Fresh PCa specimens were obtained with written informed consent through the Australian Prostate Cancer BioResource from men undergoing robotic radical prostatectomy at St Andrew’s Hospital (Adelaide, Australia) and cultured for 48 h with 17-AAG, AUY922 (500 nM each) as previously described^16^. Clinicopathological features of tumors used in this study are detailed in Table 1. Tissues were cultured at 37 °C for 48 h, then formalin fixed and paraffin-embedded for histology, snap frozen in liquid nitrogen for proteomics analysis, or stored in RNAlater for transcriptomic analysis.

**Table 1:** Pathologic characteristics of tumors used in this study.

Analysis	Patient ID	Age at RP (years)	PSA at RP (ng/mL)	Gleason Grade	Pathologic Stage
transcriptomics	1921	52	6.3	4 + 3 = 7	PT3A
transcriptomics	1963	59	12	5 + 5 = 10	PT3A
transcriptomics	1967	70	13	4 + 3 = 7	PT3B
transcriptomics	1970	56	2.4	3 + 4 = 7	PT2C
transcriptomics	2100	63	19.6	4 + 3 = 7	PT3B
transcriptomics	2117	51	8	3 + 4 = 7	PT3A
proteomics	1381	60	7.7	4 + 3 = 7	PT3B
proteomics	1411	58	5.7	3 + 4 = 7	PT2C
proteomics	1420	62	5.1	3 + 4 = 7	PT3A
proteomics	1424	66	9.1	3 + 3 = 6	PT3A
proteomics	1439	63	6.8	3 + 3 = 6	PT2C
proteomics	1455	61	5.2	5 + 4 = 9	PT3B
proteomics	1463	67	15	4 + 3 = 7	PT2C
proteomics	1490	66	6.4	4 + 3 = 7	PT3B
proteomics	1581	63	17	4 + 3 = 7	PT3A
proteomics	1596	66	6.6	4 + 5 = 9	PT3A
proteomics	1622	72	13	3 + 4 = 7	PT2C
proteomics	1628	74	7.1	3 + 4 = 7	PT2C

All tumors are adenocarcinoma of acinar type and pathologic stages are as per AJCC TNM 7th Edition.

### RNA Sequencing

Hsp90 inhibitor or vehicle treated patient derived explants (n = 6) preserved in RNAlater were homogenized with the Precellys®24 Tissue Homogenizer (Bertin Technologies, France) using 0.5 mL Precellys®24 tubes containing 1.4 mm ceramic beads and 500 mL Tri Reagent (Sigma). RNA was then extracted using a Qiagen RNeasy mini kit adapted for Tri Reagent using the manufacturer instructions. RNA underwent quality control using the Agilent Bioanalyzer (Agilent, Santa Clara, CA) and all samples submitted for sequencing had an RNA Integrity Number (RIN) ≥6. Library preparation and sequencing were performed at the ACRF Cancer Genomics Facility (SA Pathology, Adelaide, Australia). Total RNA samples were treated with Ribo-Zero to remove rRNA prior to library construction with the Illumina TruSeq Stranded RNA kit. Sequencing was performed on an Illumina HiSeq. 2500 to generate 2 × 125 bp paired-end reads. All samples were pooled and run across 3 lanes. Reads were mapped to the human genome (Version hg19) with Tophat2 (Version 2.0.4)^58^. Mapped reads were assembled with CuffLinks (Version 2.2.1)^59^. Differential expression (DE) analysis was conducted in R using edgeR package (version 3.18.1). After quality control (QC: mean count per million ≥10 for at least 3 samples), the initial number of 58,051 genes was reduced to 22,818 genes. Before DE testing, we fitted the quasi-likelihood negative binomial generalized log-linear model out after QC data using a robust version of edgeR’s glmQLFit function. Next, due to greatly different potency of AUY922 and 17-AAG inhibitors, we used two different DE testing strategies. Firstly, AUY922 vs. DMSO and AUY922 vs. 17-AAG comparisons were based on edgeR’s glmTreat function with the null absolute fold change set to 1.8, accepting genes with BH adjusted P-values less than 5% as DE. Secondly, to detect a much milder effect of the 17-AAG inhibitor, we applied edgeR’s glmQLFTest function to the 17-AAG vs. DMSO comparison, accepting genes with BH adjusted P-values less than 5% and absolute fold change greater than 1.2 as DE. Enrichment barcode plots and heatmaps were generated by barcodeplot and heatmap.2 functions from limma and gplots R packages, respectively.

### Proteomics Analysis by LC-MS

Snap frozen PDEs (n = 12 patients) were homogenized using the Precellys®24 Tissue Homogenizer (Bertin Technologies, France) and 0.5 mL Precellys®24 tubes containing 1.4 mm ceramic beads and 120 µL ice cold TUC4% buffer (7 M Urea, 2 M Thiourea, 4% CHAPS, 30 mM Tris, pH 8.5). Tissue lysate was transferred to 1.5 mL Eppendorf tubes and centrifuged at 10,000 rpm for 10 min at 4 °C before supernatant removed and stored at −80 °C. Frozen tissue lysates were provided to the Adelaide Proteomics Centre (University of Adelaide, Adelaide, Australia) where 2D-DIGE proteomics analysis performed as described in^60^ with the following modifications: Individual explant samples containing 50 μg of protein were labelled with Cy3 and Cy5. Separate internal standards were prepared by pooling 25 µg of protein from each sample and labelled with Cy2. The dye/protein ratio for all samples was 200 pmol/50 μg. Pharmalyte 3–10 (GE Healthcare) was added to all samples prior to isoelectric focusing, which was performed on 24 cm 3–10 non-linear (NL) immobilized pH gradient (IPG) strips (Biorad). Proteins were focused for 27,000 Volt-hours at 8,000 V. SDS-PAGE in the second dimension was carried out using 12.5% 2DGel DALT NF pre-cast polyacrylamide gels (Gel Company). Gels were scanned using an Ettan DIGE Imager (GE Healthcare) at 100 μm resolution and analysis undertaken using DeCyder 2D software (version 7, GE Healthcare). In Difference In-gel Analysis (DIA) mode, spot detection was performed based on an estimated 5,000 spots. Exclusion filters were set to reject spots with a slope of >2, an area of <400, a volume of <30,000 and a peak height of <300 and >100,000. Each gel image was processed separately in the Differential In-gel Analysis (DIA) module of DeCyder for spot detection prior to export to the Biological Variation Analysis (BVA) module. Protein abundance in AUY922 treated PDEs was subjected to statistical comparison with its vehicle and 17-AAG treated counterpart to detect spots that are differentially expressed using paired two-tailed Student’s t-test. Spots that returned a p-value of <0.05 were considered differentially expressed.

### Extraction of protein from cell lines

PCa cells were seeded in 6-well culture plates [1.5 × 10^5^ cells per well] and cultured for 24 h prior to treatment as indicated. Protein lysates were collected from LNCaP and PC-3 cells following 48 h treatment with vehicle or the indicated doses of AUY922 or 17-AAG. Cells were washed in PBS and lysed directly from 6-well plates using RIPA buffer (10 mM Tris, 150 mM NaCl, 1 mM EDTA, 1% TX-100) containing protease inhibitor cocktail (Sigma-Aldrich). Cells were scraped and collected into a 1.5 mL Eppendorf tube and homogenized with a 26 G needle prior to centrifugation at 10,000 rpm for 10 mins at 4 °C. Supernatant was collected and stored at −80 °C.

### Western Blotting

Whole cell protein lysates were prepared as described above and analyzed by Western blot as described previously^61^. Proteins were digitally imaged using the ChemiDoc™ XRS (BioRad). Full length blots can be found in Supplementary Figure 6.

### Cell Motility

LNCaP and PC-3 PCa cells grown in RPMI with 10% or 5% FBS respectively, were seeded in 24-well Image Lock plates (Essen BioScience) at a density of 4 × 10^4^ cells per well, and cultured for 24 h prior to treatment. The seeding media was then replaced with growth media containing either vehicle (DMSO), 17-AAG or AUY922 at doses equivalent to IC50 (25 nM AUY922, 50 nM 17-AAG) or 2x IC50 (50 nM AUY922, 100 nM 17-AAG). Cells were placed in an IncuCyte (Essen BioScience) live cell analysis system, and images of the cells were captured using 20x magnification at 10 min intervals for 48 h. The image sets from each treatment were imported into ImageJ v1.49t, where the plugin MTrackJ was used to track the movement of individual cells over time. Tracking of a cell would cease if the cell a) divided b) died c) lifted from the plate or d) moved out of view. Cell motility was calculated as cell track length (pixels) per minute.

### Immunofluorescence

Cells were seeded in 6-well plates [1 × 10^5^ cells per well] onto circle glass cover slips of 0.6 µm thickness (Thermo Fisher) and allowed to attach for 24 h. Cells were treated for 48 h with AUY922 (25 nM), then fixed in 4% paraformaldehyde for 20 min and permeabilized using 0.1% TritonX-100 for 10 min. Cells were incubated in blocking buffer (10% serum in PBS) for 30 min at room temperature before immunostaining with primary antibody for 2 h at room temperature followed by appropriate fluorophore-conjugated secondary antibody (anti-goat Cy3, anti-mouse Alexa Fluor 594, anti-rabbit Alexa Fluor 488; 1:250) for 1 h at room temperature in the dark. Cover slips were mounted using Prolong Gold™ Antifade Mountant Media containing DAPI and cured for 48 h before imaging on a Leica SP8 confocal microscope.

The cellular tubulin polymer mass was measured based on the mean α-tubulin fluorescence intensity as described in^62^. Optical 96-well plates (ibidi) were coated with poly-L-ornithine (Sigma-Aldrich), and LNCaP cells [5 × 10^3^ cells per well] were seeded and allowed to attach for 24 h. Cells were treated for 48 h with the indicated doses of inhibitors (3 wells/treatment) after which cells were fixed with ice-cold methanol for 3 min, incubated in blocking buffer [2% bovine serum albumin (Sigma-Aldrich) in TBS with 0.1% Triton X-100] for 30 min at room temperature before immunostaining for 1 h at room temperature with primary antibody against α-tubulin (DM1A, ab7291, Abcam, 1:500). Cells were washed twice in TBS-T and incubated with the appropriate secondary, fluorophore-labelled antibody for 1 h at room temperature in the dark. DNA was counterstained with 1 µg/mL DAPI [4′,6-diamidino-2-phenylindole; Sigma-Aldrich]. Methanol fixation and permeabilization with Triton X-100 were performed to wash out soluble tubulin subunits^63,64^. Cells were imaged on an INCell 2200 automated imaging systems (GE Healthcare) at 20x magnification. Image segmentation of ~4,500 cells/treatment and quantitation of mean α-tubulin fluorescence intensity were performed with CellProfiler software (Broad Institute, Cambridge, USA)^65^. All data points were performed in technical triplicates and are reported as the mean ± SD.

### Cellular Fractionation

Cells were seeded in 6-well plates [1.0 × 10^5^ cells per well] and allowed to attach for 24 h. Cells were treated for 48 h with the indicated dose of inhibitor, then trypsinized and an equal number of cells collected from each sample for fractionation. Cells were resuspended in 300 μL of lysis buffer 1 (50 mM Hepes-KOH pH 7.5, 140 mM NaCl, 1 mM EDTA, 10% glycerol, 0.5% NP-40, 0.25% TX-100) containing protease inhibitor cocktail (Sigma-Aldrich), and 100 μL of suspension was collected as whole lysate. Samples were centrifuged at 9,300 rcf for 10 sec and 100 μL supernatant collected as cytoplasmic lysate. Remaining cells were washed in lysis buffer 2 (10 mM Tris-HCl pH 8.0, 200 mM NaCl, 1 mM EDTA, 0.5 mM EGTA) containing protease inhibitor cocktail (Sigma-Aldrich), and resuspended in 100 μL lysis buffer 3 (10 mM Tris-HCl pH 8.0, 100 mM NaCl, 1 mM EDTA, 0.5 mM EGTA, 0.1% Na-deoxycholate, 0.5% N-lauroylsarcosine) containing protease inhibitor cocktail (Sigma-Aldrich). All samples were sonicated at high intensity for 10 × 30 sec with a 1 min rest on ice followed by centrifugation at 15,700 rcf for 10 min.

### Enzyme-linked immunosorbent assay (ELISA)

LNCaP cells were seeded in 6-well culture plates [1.5 × 10^5^ cells per well] and allowed to adhere for 24 h. Cells were treated for 48 h in FN-1 free HPC1 media (Sigma-Aldrich) with vehicle or AUY922 (25 nM). Culture medium was collected from the cells and gently centrifuged to generate cell-free media, which was immediately snap frozen. ELISA plates (Jomar Bioscience cat# BMS2028) were assayed according to the manufacturer’s instructions. Cells remaining in the plate were trypsinized and total cell number for each sample was determined to calculate the amount of protein secretion per cell.

### *In vitro* tubulin polymerization assay

Microtubule assembly was studied using the CytoDYNAMIX Screen kit (BK006P; Cytoskeleton Inc., Denver, CO, USA). Purified porcine tubulin protein (>99% purity) was resuspended in G-PEM buffer containing 80 mM PIPES, 2 mM MgCl_2_, 0.5 mM EGTA, 1 mM GTP (pH 6.9) and 15% glycerol in the absence or presence of indicated compounds at 4 °C. The mixture was transferred into a pre-warmed 96-well plates, and polymerization was monitored (absorbance) using a FLUOstar Omega plate reader (BMG LABTECH) at 340 nm every 1 min for 60 min at 37 °C.

### Exosome preparation and NanoSight particle tracking analysis

Cells (LNCaP, PC-3 and 22Rv1) seeded at 60–80% confluency were cultured with DMSO control, 25 nM or 50 nM AUY922 in exosome-depleted media for 48 h. Medium was collected and exosomes were isolated as described previously^22^. The concentration of exosomes released was quantified using Nanoparticle Tracking Analysis (NTA) software version 2.3 on the NanoSight LM10-HS10 system (NanoSight Amesbury, UK). When quantifying exosomes released, samples were diluted in exosome-free pre-filtered PBS to obtain measurable concentrations between 0.5 × 10^8^ and 5 × 10^9^ particles/mL. For each sample dilution, three “real-time” readings were taken, and the data presented is the average +/−SEM of these 3 readings.

### Transwell invasion assay

3D invasion assays were conducted using 24-well Falcon BioCoat Matrigel invasion chambers (#FAL354480, *In Vitro* Technologies, VIC, Australia). PC-3 cells (5 × 10^5^) were transfected using 5 µM RNAiMax according to the manufacturer’s instructions with 5 pmol siRNA against FN1 (Dharmacon J-009853-07, J-009853-06), or non-targeting siRNA (Dharmacon D-001810-01) as a negative control for 48 h before harvesting for the invasion assay. In order to evaluate the effect of AUY922 and pUR4 on cell invasion, PC-3 cells (3 × 10^5^) were seeded in 6-well plates and allowed to adhere overnight. Cells then were treated with either AUY922 (25 and 50 nM), or pUR4 (500 nM) for 48 h before harvesting for the invasion assay. DMSO (0.5%) or the control peptide III-11C (500 nM) were used as negative controls. Treated or siRNA-transfected cells were seeded into the upper chamber of the Transwell at a density of 1 × 10^5^ cells/well in serum-free medium. The bottom chamber was filled with 650 µL of RPMI-1640 medium containing 5% FBS. The inserts were incubated at 37 °C for 48 h. Non-migrated cells were removed from the upper surface of the membrane with a cotton swab, and the membranes were washed three times with PBS. Migrated cells remaining on the bottom surface were fixed in formalin for 20 min, washed and stained with crystal violet for 30 min. Five random fields for each membrane were photographed using the Axio Scope A1 Fluorescent Microscope (Zeiss), and the number of migrated cells was counted manually and presented as percent of control cells ± SEM.

### Statistical Analysis

One-way ANOVA with Dunnet’s multiple comparisons test (GraphPad Prism 7 software, GraphPad Software) was used for statistical analysis in this study (ns = non-significant, *P < 0.05, **P < 0.01, ***P < 0.001, ****P < 0.0001).

### Data Availability

The datasets generated during and/or analysed during the current study are available from the corresponding author on reasonable request.

## Electronic supplementary material

Supplementary Figures

Supplementary Dataset
