# Supplementary material for: Dysregulated fibronectin trafficking by Hsp90 inhibition restricts prostate cancer cell invasion
Source: Sci Rep. 2018 Feb 1;8:2090. doi: 10.1038/s41598-018-19871-4 (PMC5794796; doi:10.1038/s41598-018-19871-4)

Dysregulated fibronectin trafficking by Hsp90 inhibition restricts prostate cancer cell invasion.

Heather K. Armstrong[1,2]*, Joanna L. Gillis[1,2*], Ian R. D. Johnson[3], Zeyad D. Nassar[1,2], Max Moldovan[2], Claire Levrier[4], Martin C. Sadowski[4], Mei Yieng Chin[5], Emma Tomlinson Guns[5], Gerard Tarulli[1,6], David J. Lynn[2,7], Douglas A. Brooks[1,3], Luke A. Selth[1,6], Margaret M. Centenera[1,2*] and Lisa M. Butler[1,2*].

Supplementary Data

Proliferation: RNA-seq cohort (n=6)

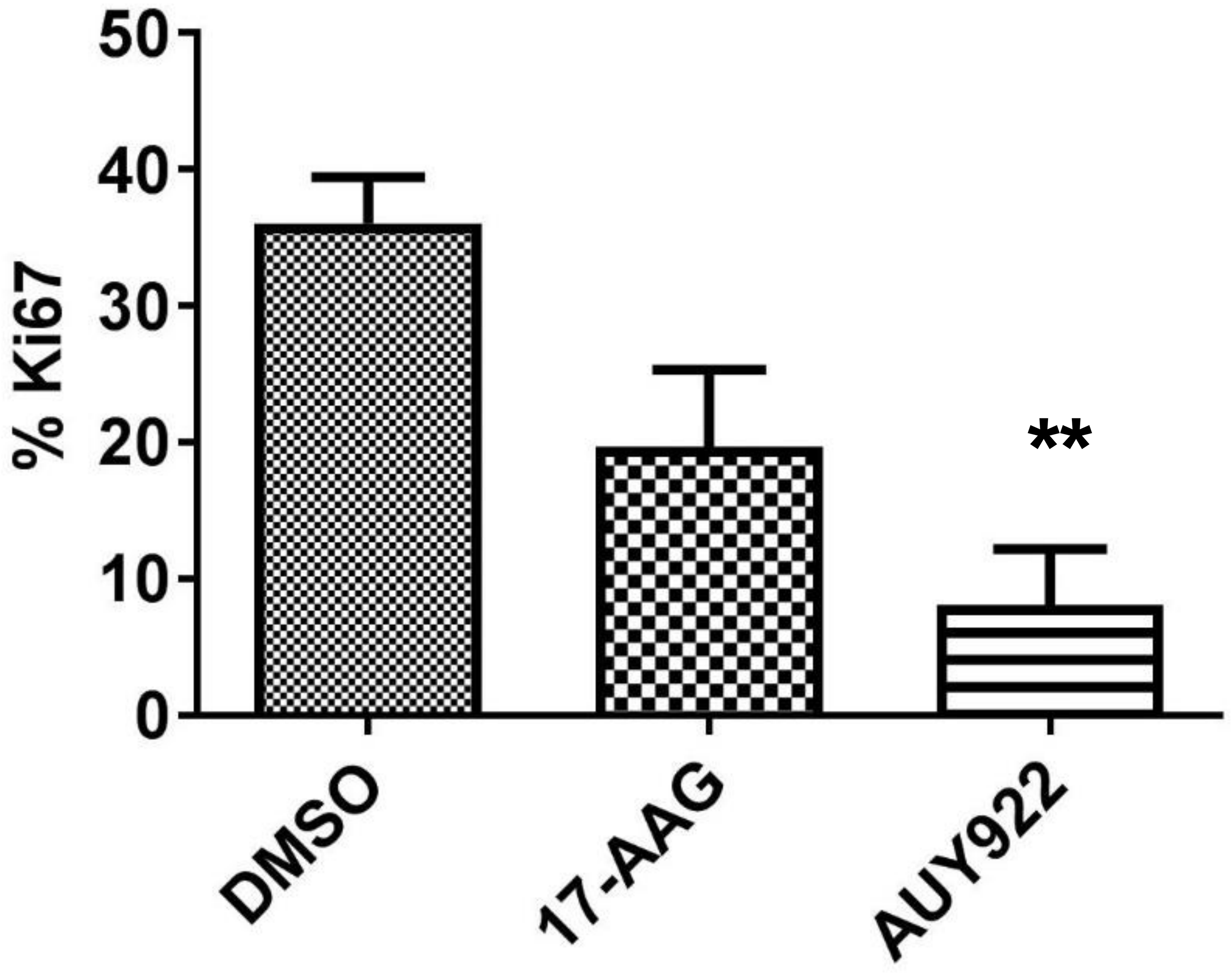

Proliferation: proteomics cohort (n=12)

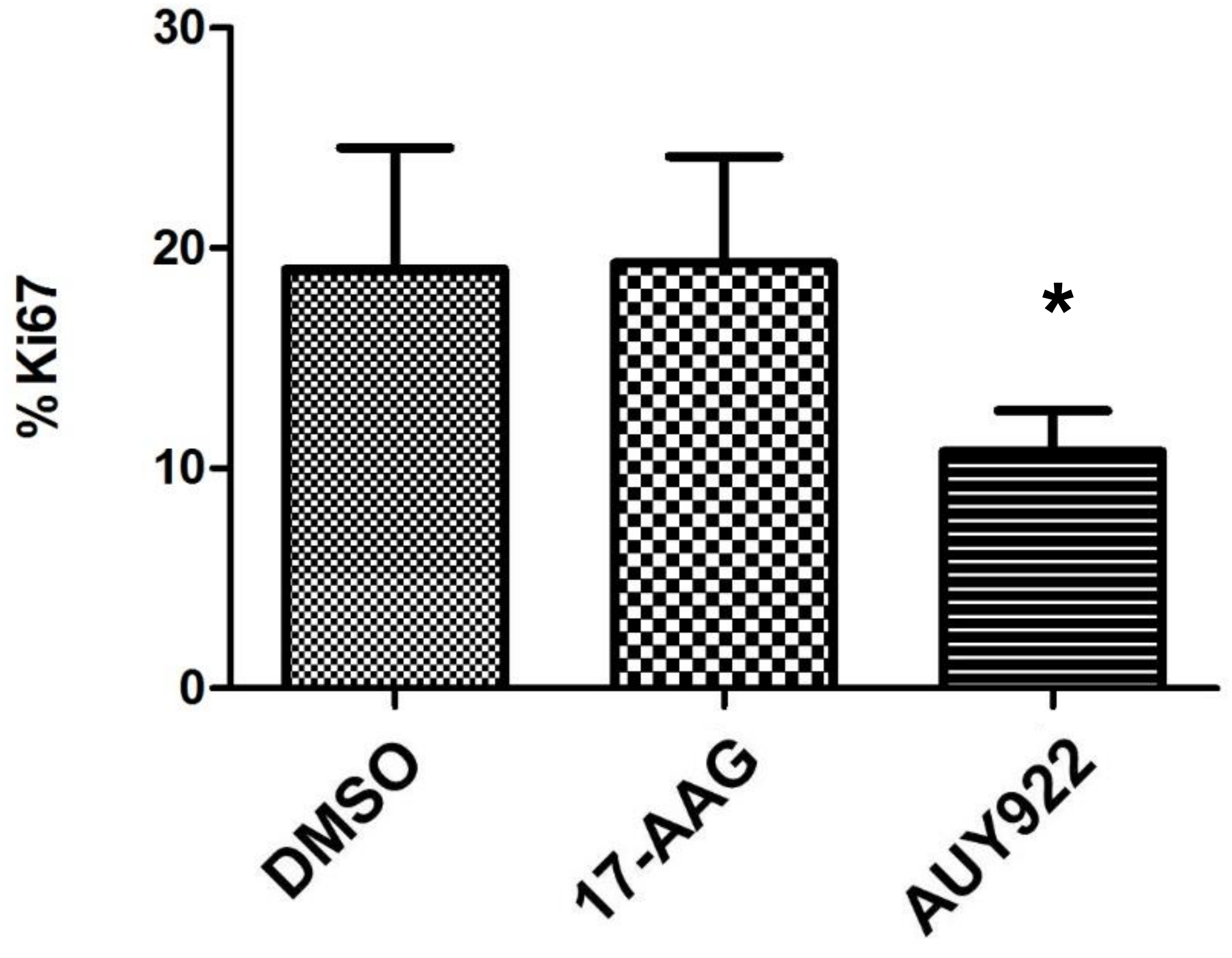

Supplementary Figure 1: Proliferative effects of 17-AAG and AUY922 in patient-derived prostate tumor explants cultured for 48h, measured as Ki67 antigen positivity.

Armstrong et al, Supplementary Figure 2

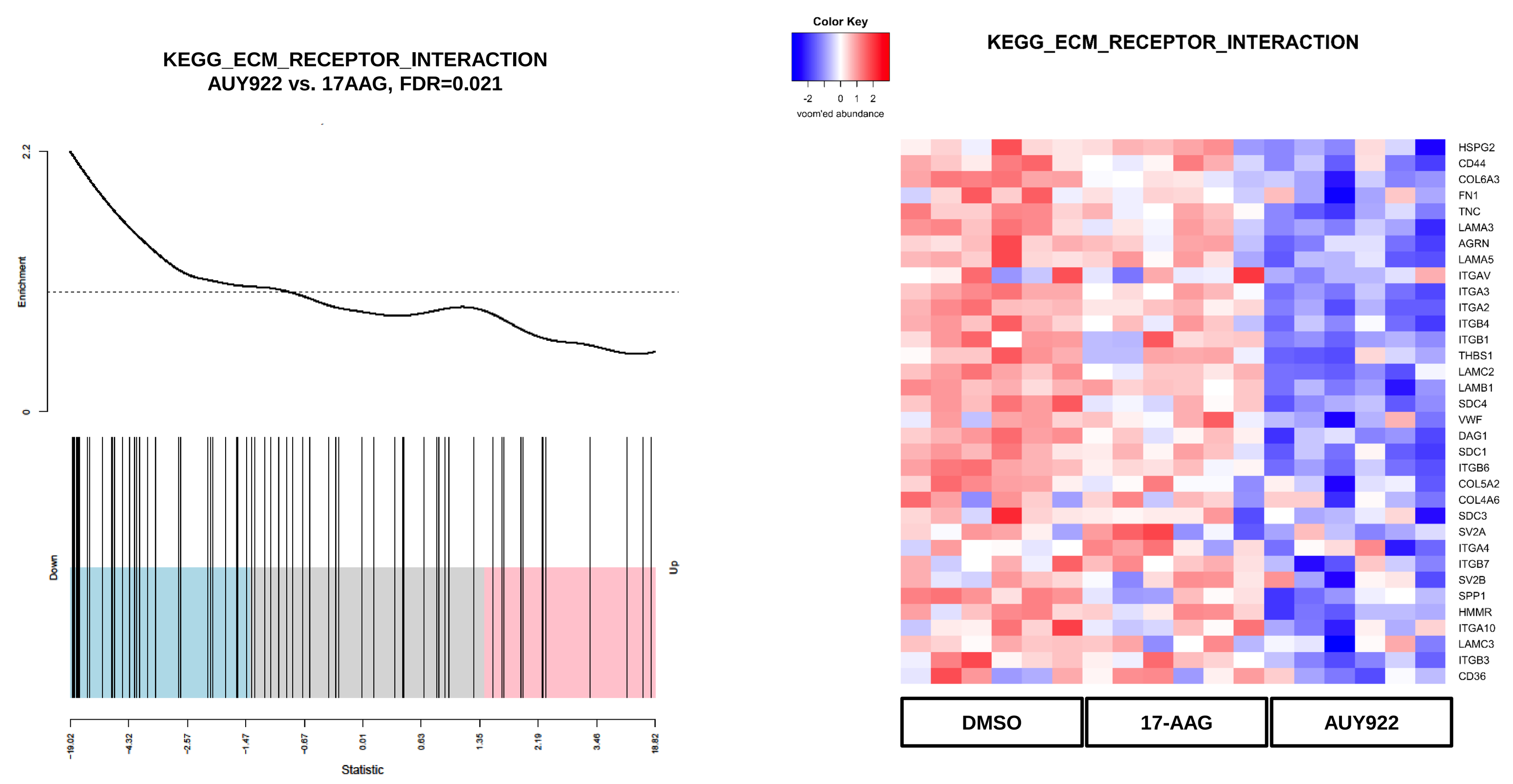

Supplementary Figure 2: A barcode plot and heatmap of RNA-seq gene expression data from extracellular matrix interactions pathway for individual explants is shown.

Armstrong et al, Supplementary Figure 3

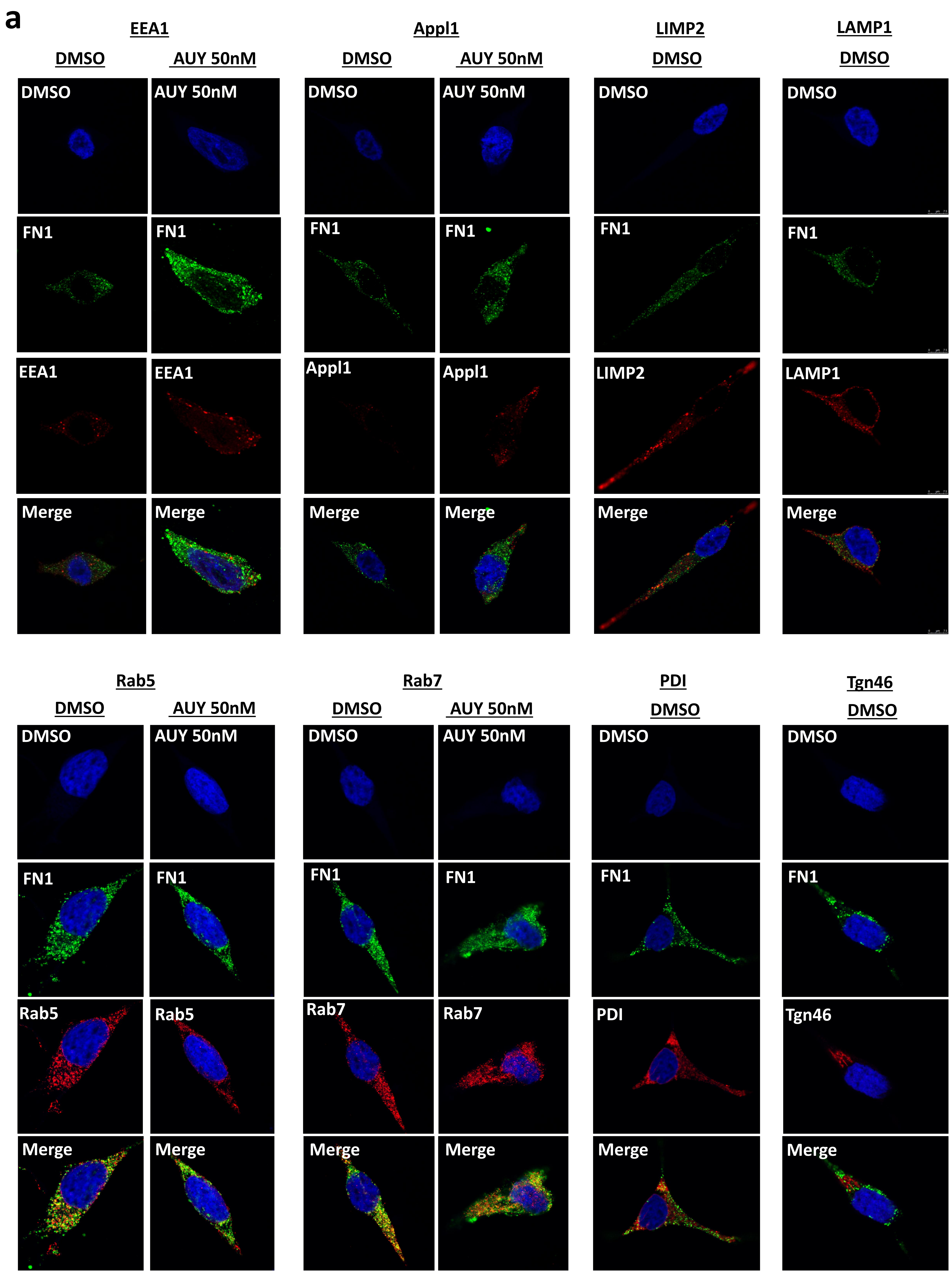

Supplementary Figure 3: Colocation of FN1 with markers of vesicles including endosomes (EEA1, Appl1, Rab5, Rab7, Rab11), lysosome (LIMP2, LAMP1), endoplasmic reticulum (PDI) and Golgi (Tgn46) was examined by fluorescence microscopy in LNCaP cells treated as indicated for 48 h and probed for vesicle markers (red) and FN-1 (green) and DAPI mount media (blue). Images were taken using Leica SP8 confocal microscope at 63x magnification.

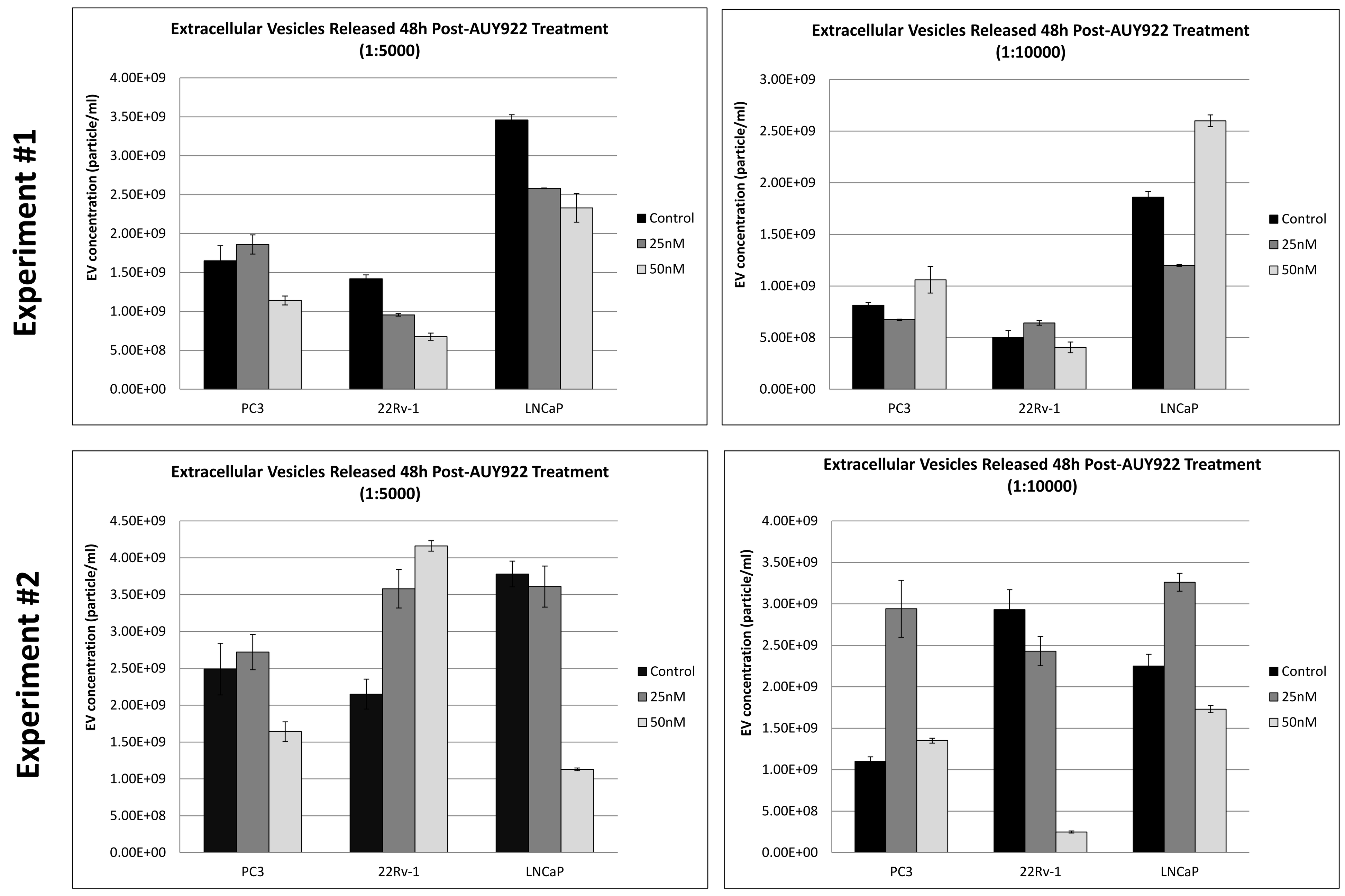

Supplementary Figure 4: Exosome release from PCa cell lines cultured in the absence or presence of AUY922. Cells (LNCaP, PC-3 and 22Rv1) were cultured with DMSO control, 25nM or 50nM AUY922 in exosome-depleted media for 48h. Medium was collected and exosomes were isolated as described previously [20]. The concentration of exosomes released was quantified using Nanoparticle Tracking Analysis (NTA) software version 2.3 on the NanoSight LM10-HS10 system.

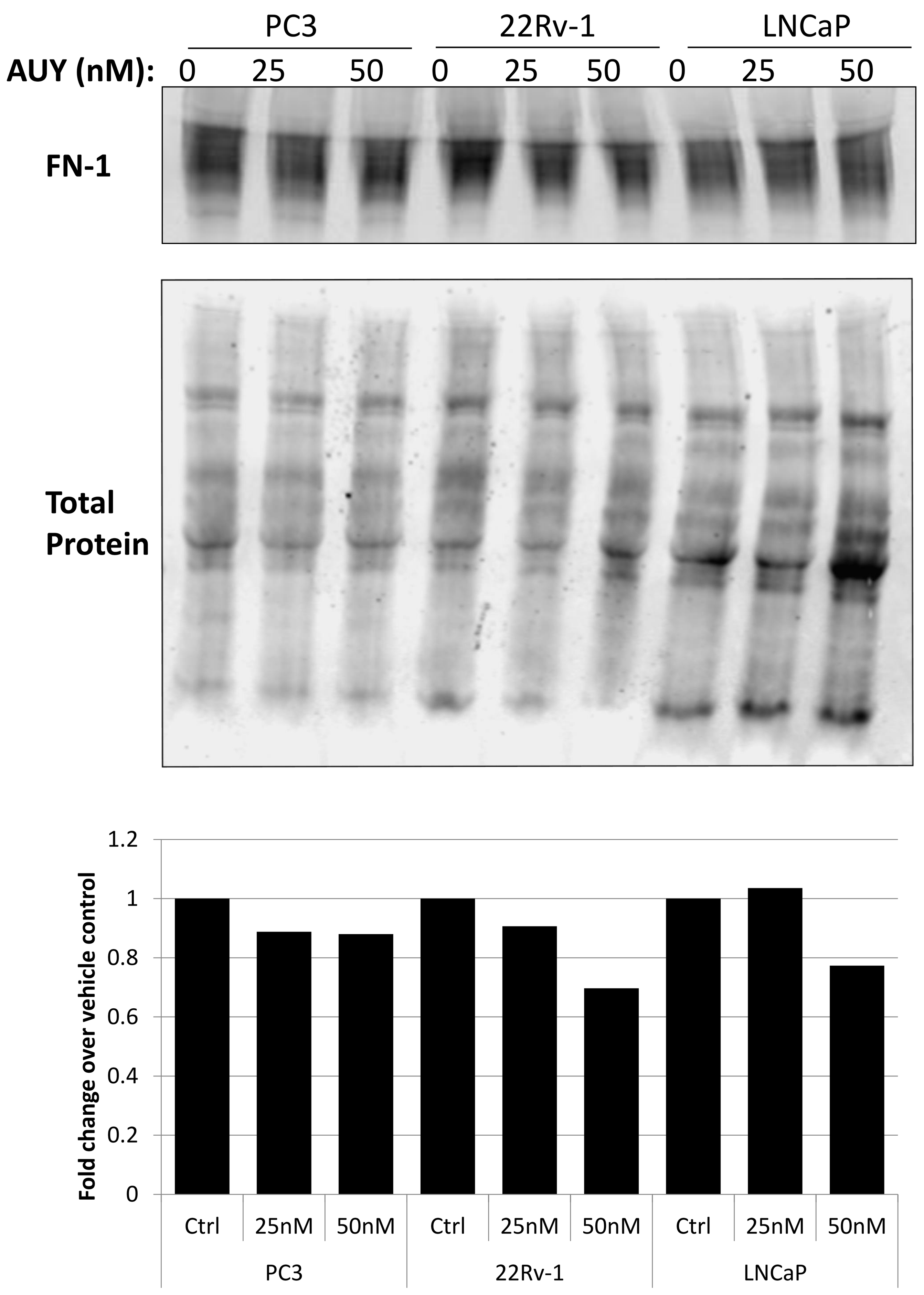

Supplementary Figure 5: Western blot and densitometr analysis of FN-1 expression in PC3, 22Rv-1 and LNCaP prostate cancer cells treated as indicated for 48 h. Total protein was used as loading control. FN-1 expression in treated samples is presented as fold change over vehicle treatment.

Armstrong et al, Supplementary Figure 6. Full western blot images

Patient-derived explants

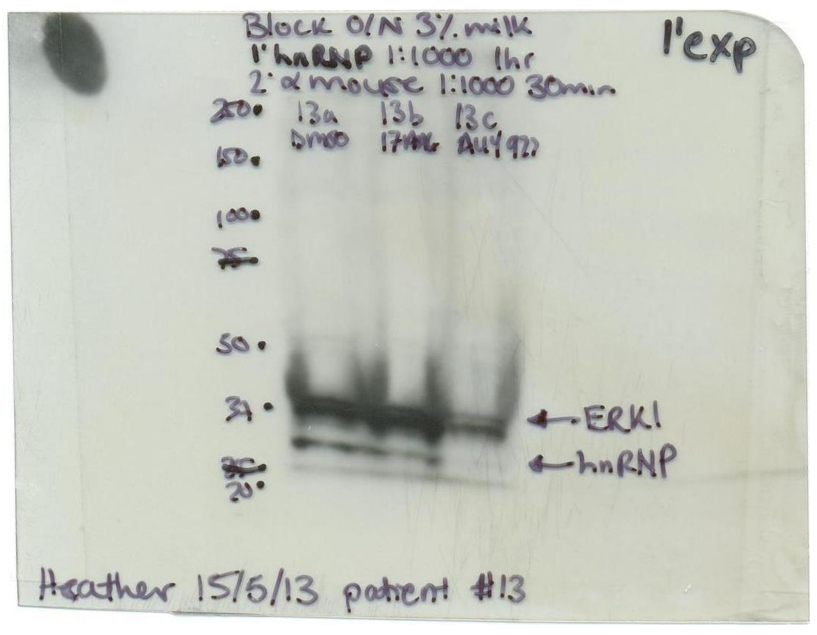

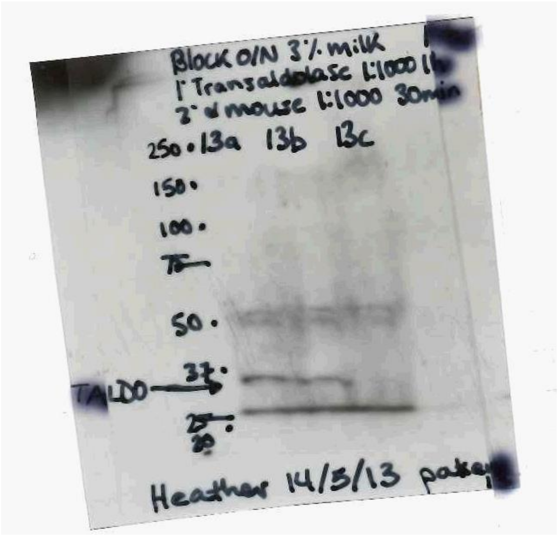

LNCaP Cell Line

HSP70

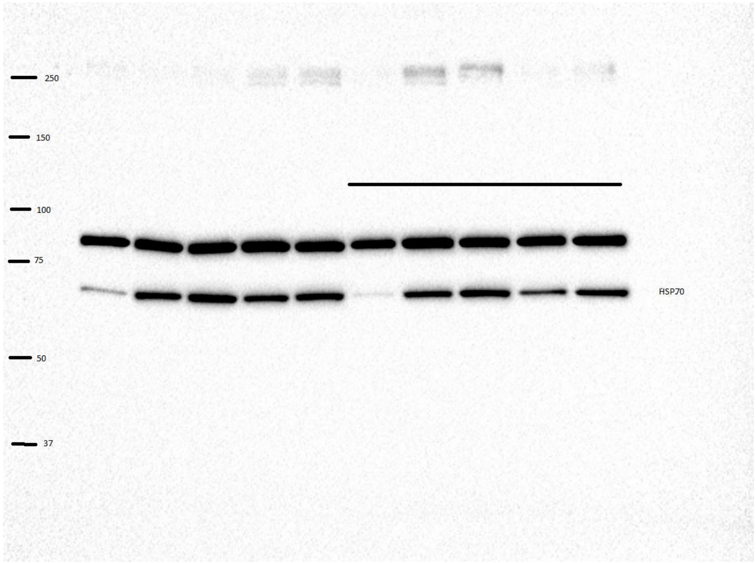

FN1

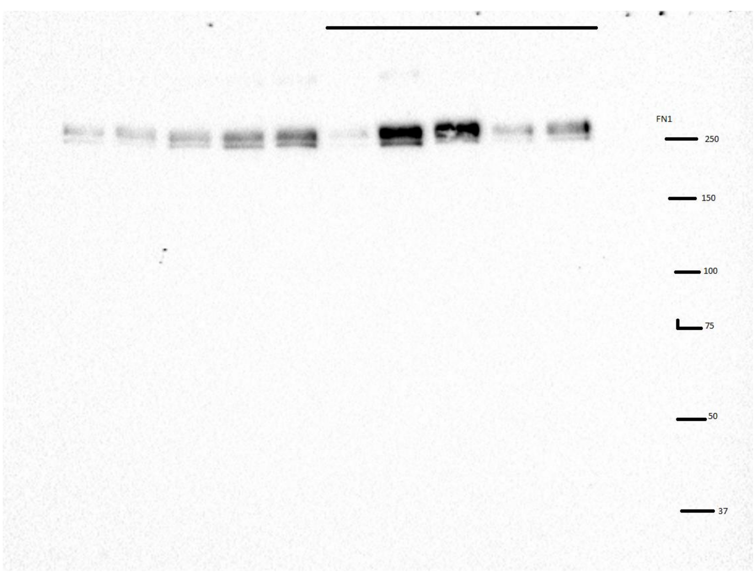

TUBULIN

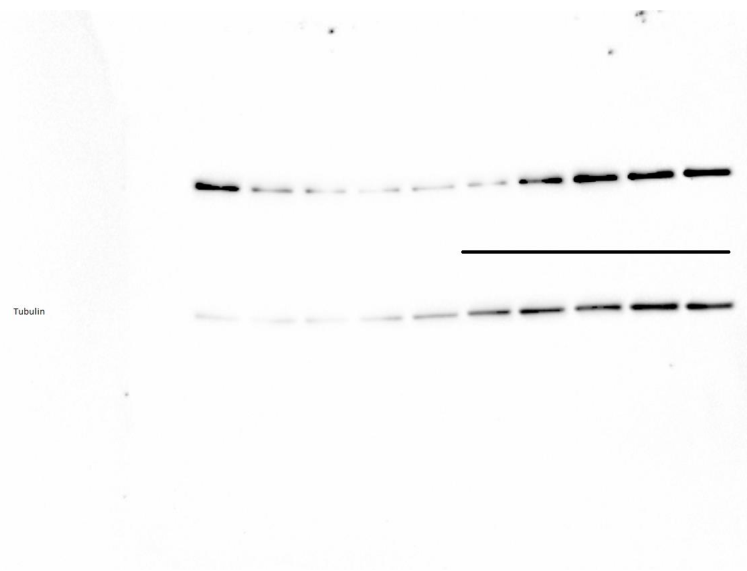

ACTIN

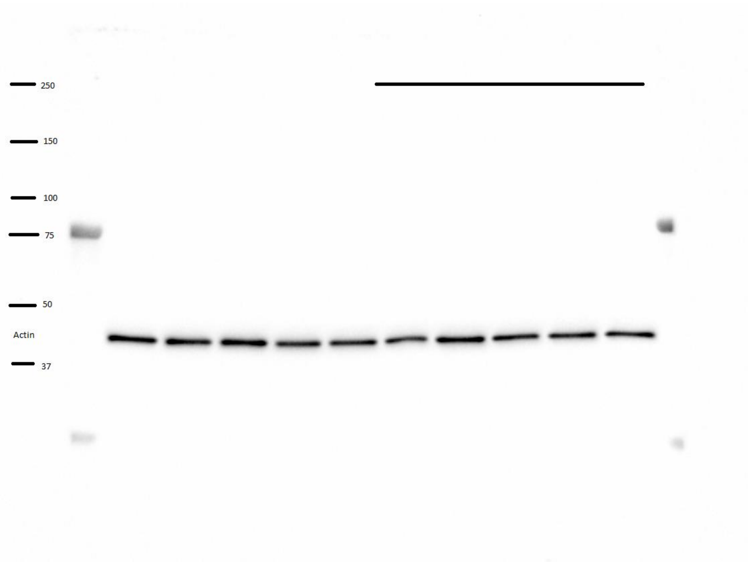

MYOSIN

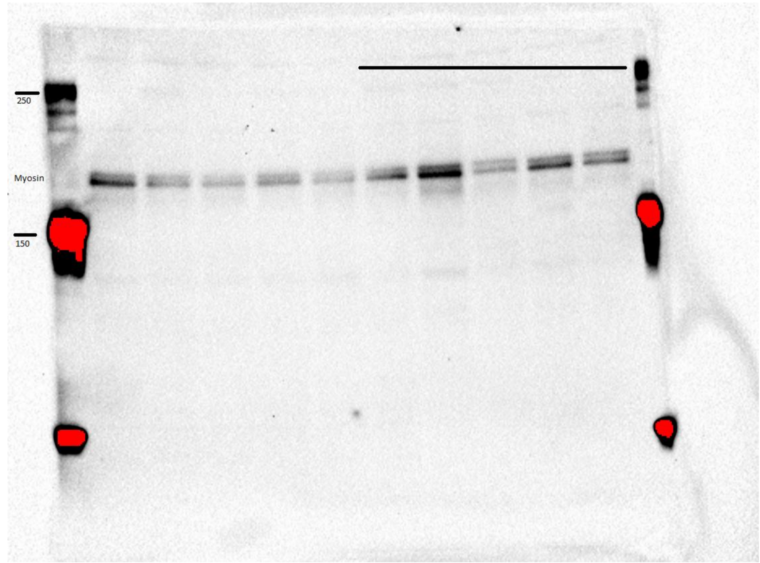

E-CADHERIN

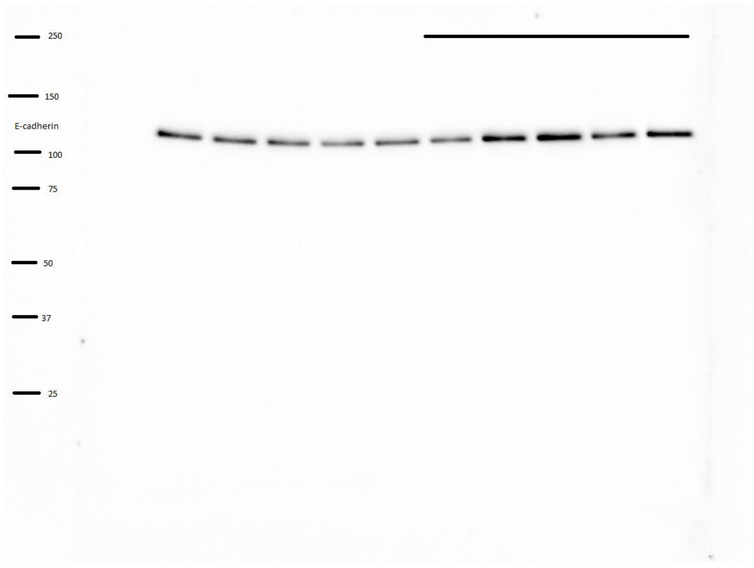

HSP90

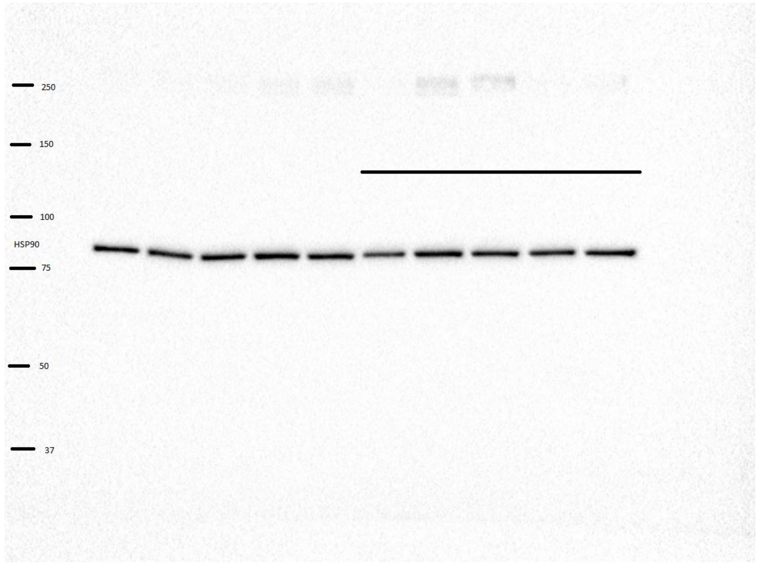

PC3 Cell Line

HSP70

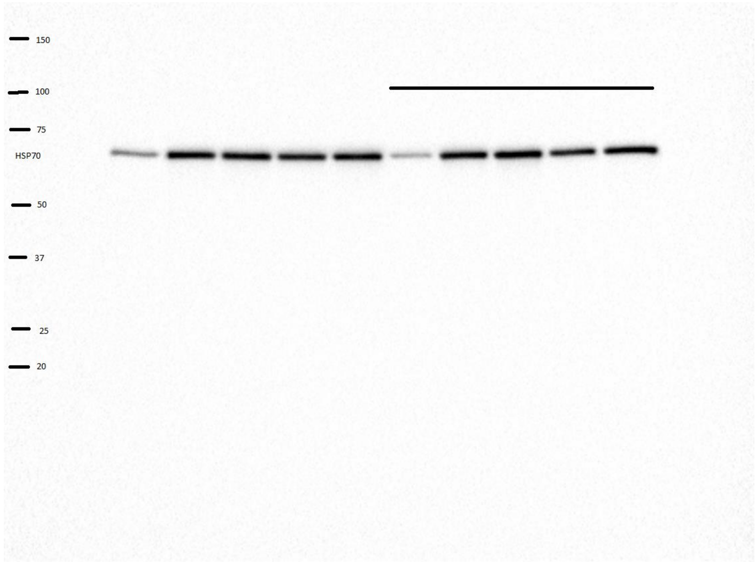

FN1

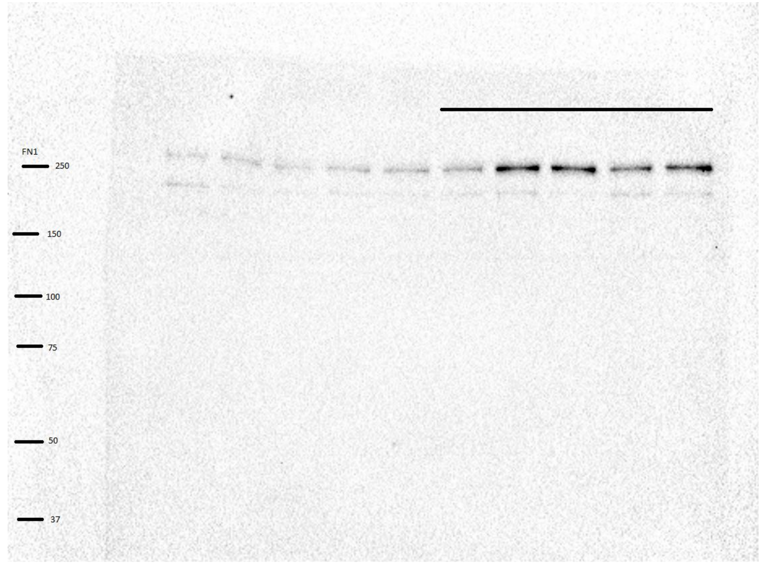

TUBULIN

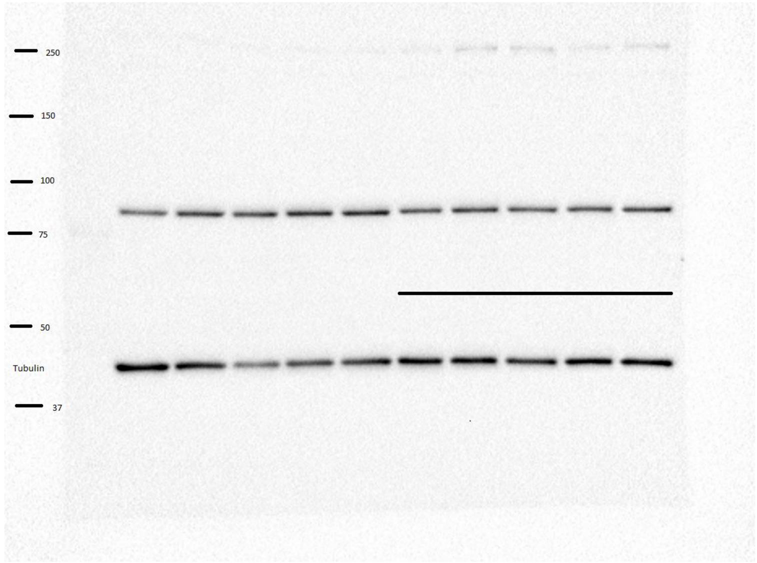

ACTIN

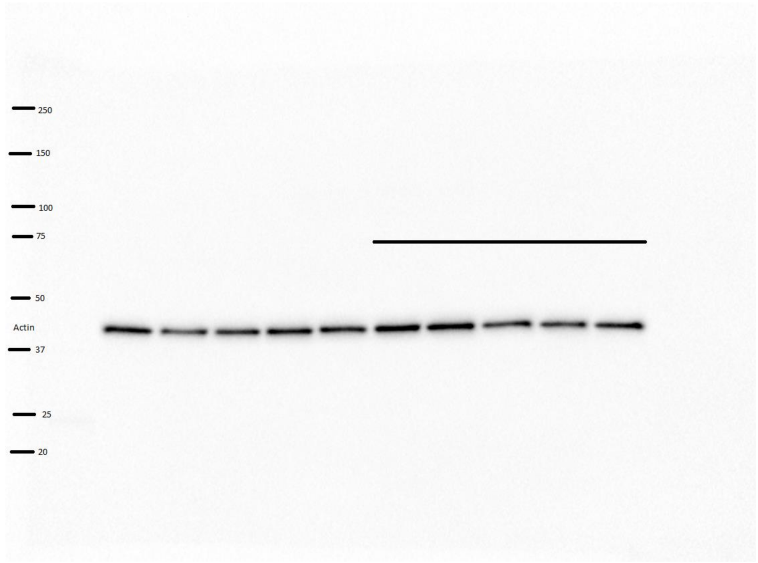

MYOSIN

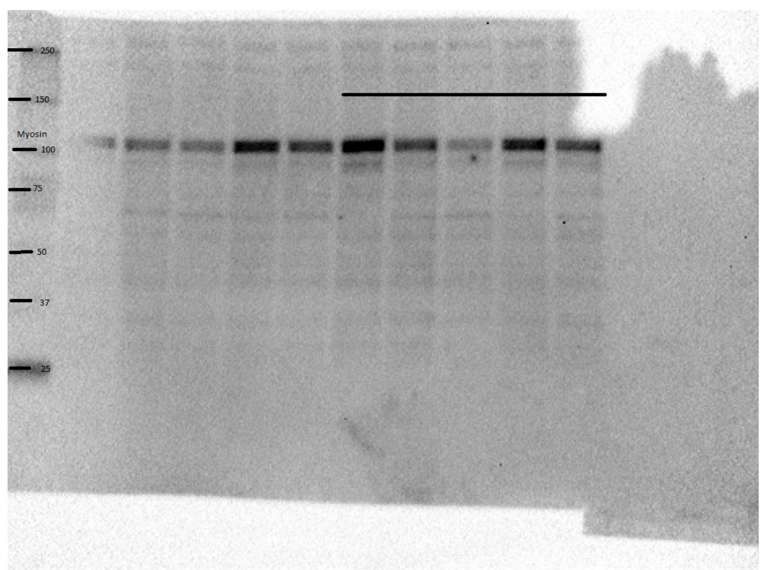

HSP90

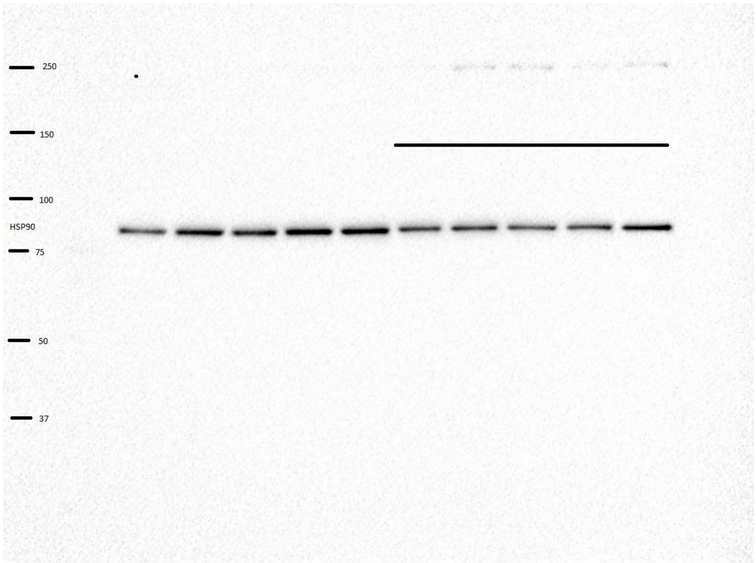

Armstrong et al, Supplementary Figure 6. Full western blot images

LNCaP Fractionation

FN1

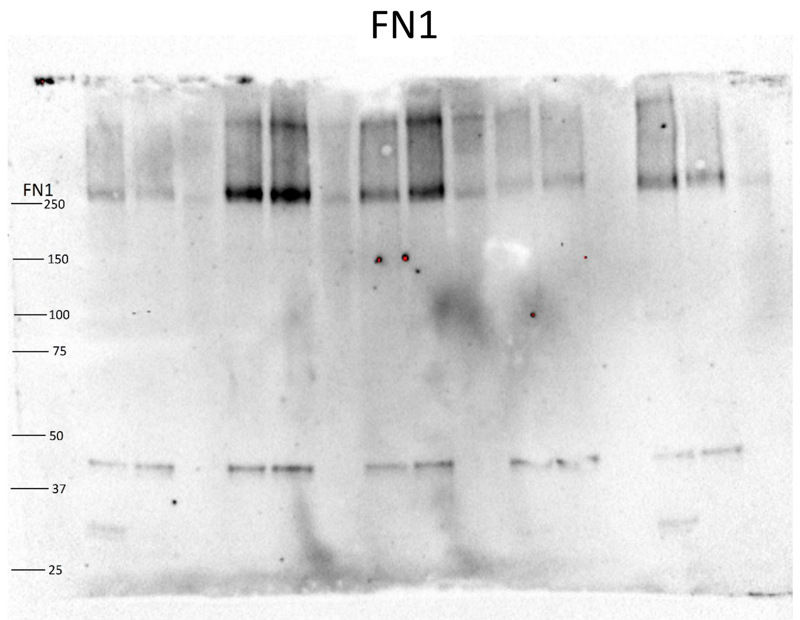

MEK1

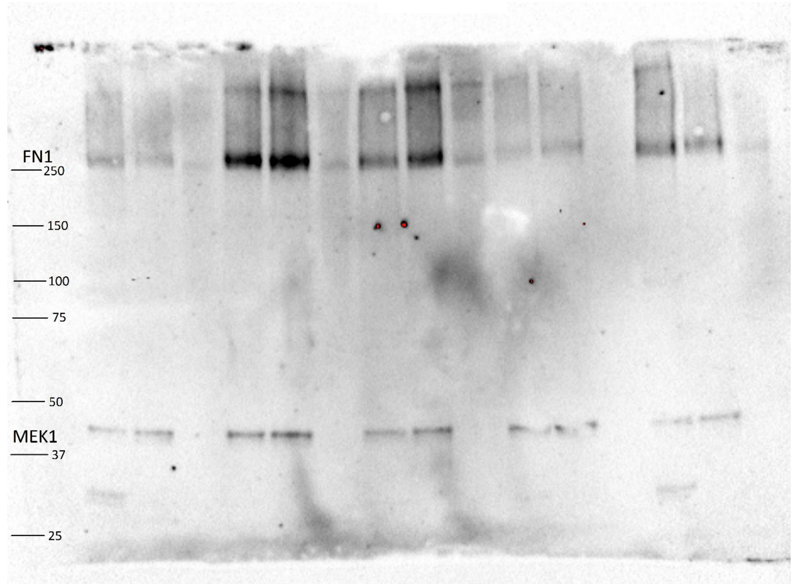

H3

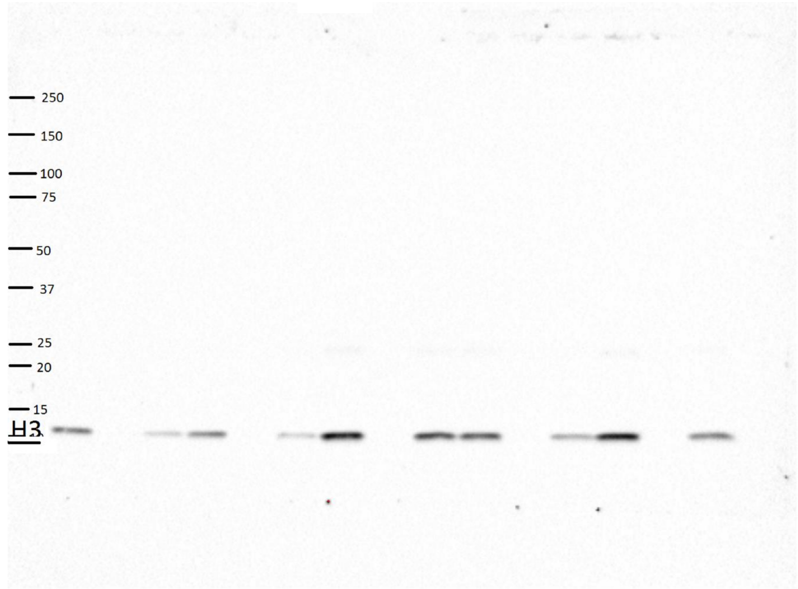

HSP90

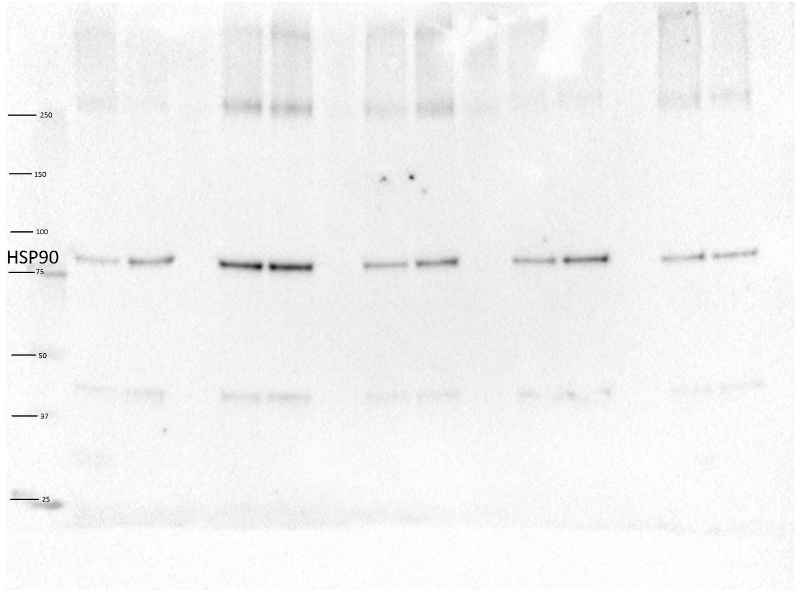

FN1 Knockdown

FN1

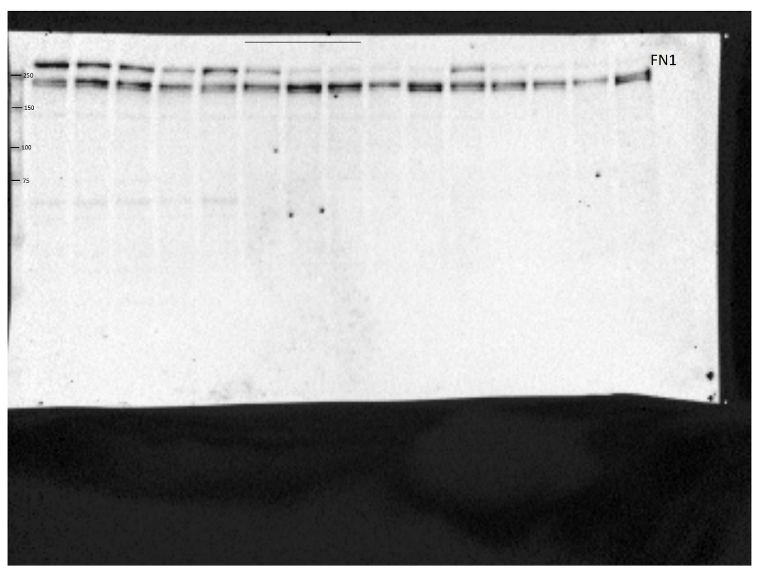

HSP90

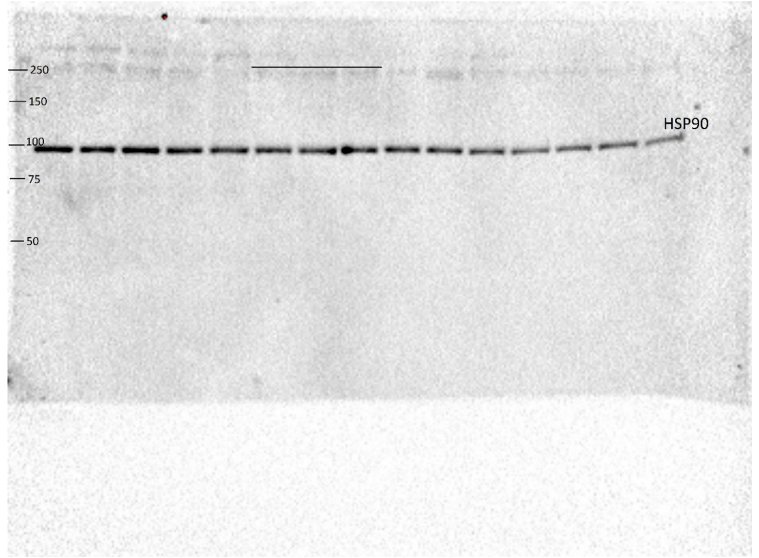

Supplement: Supplementary file 1 — Supplementary Figures [file 41598_2018_19871_MOESM1_ESM.pdf]
